# Assessment of suitability evaluation for *Ficus altissima* blume ancient trees in different climatic environments in Guangxi, China

**DOI:** 10.3389/fpls.2025.1613723

**Published:** 2025-07-07

**Authors:** Hongliang Gu, Yuxia Chen, Qinqin Zhang

**Affiliations:** School of Resources and Environment, Anqing Normal University, Anqing, China

**Keywords:** *F. altissima*, ancient trees, species distribution models, Maxent, Guangxi Zhuang autonomous region

## Abstract

**Introduction:**

The Guangxi Zhuang Autonomous Region of China is home to numerous Ficus altissima Blume (*F. altissima*) ancient trees over 300 years old, which hold significant cultural, ecological, and scientific research value. However, little is known about their current and future distribution suitability or growth trends.

**Methods:**

To address this gap, using machine learning and species distribution models, we analyzed their distribution patterns and habitat suitability changes under current and future climate scenarios, incorporating 33 climatic, topographic, and soil-related driving factors.

**Results:**

Our findings reveal that the region hosts 514 ancient *F. altissima* trees aged over 300 years, 114 of which exhibit declining or endangered growth conditions. The spatial distribution of these trees is highly discrete, influenced by topographical constraints and intraspecific competition. Over 45% are found at elevations between 80 and 150 meters. Jackknife analysis identified the mean temperature of the wettest quarter (bio8) as the most critical factor affecting their distribution (77.6% cumulative contribution when combined with temperature seasonality, bio4). Specifically, regions with bio8 < 26°C and bio4 > 625°C are unsuitable for *F. altissima* (LOV < 0.085). Additionally, tree size varies by habitat due to competition, with ancient trees in flat areas exhibiting larger average crown widths. The accuracy of the Maxent model is superior to models such as BIOCLIM and GLM. Compared to current, under the SSP1-2.6 and SSP5-8.5 climate scenarios, the moderately suitable distribution area expanded by 1.47% and 0.89%, respectively (averaging 5.53% across four time periods), while the highly suitable area decreased by 0.04% and 0.21%. These results provide valuable insights for the conservation and sustainable utilization of ancient *F. altissima* trees.

## 1 Introduction

Ancient trees (≥100 years old) serve as living archives of historical and ecological change, playing a vital role in maintaining ecosystem stability, preserving biodiversity, and safeguarding cultural heritage (Mahmoud et al., 2015; Yan, 2023). However, their survival is increasingly threatened by urbanization, climate change, and environmental degradation (Xie et al., 2024b), contributing to a global decline in ancient tree populations (Lindenmayer et al., 2012; Huang et al., 2023). Balancing human development with the conservation of natural resources, including forests, remains a critical challenge of the 21st century (Kozlowski and Song, 2022), necessitating more effective strategies for protecting and sustainably utilizing these irreplaceable natural assets (He et al., 2024). Existing research has explored various aspects of ancient trees, including status assessments, conservation strategies, revitalization techniques, management systems, regional distribution patterns, and cultural-scientific value (Jim and Zhang, 2013; Zhang et al., 2017). Yet, systematic investigations into species-specific climate-environment relationships and future habitat suitability remain scarce, particularly at localized scales. Understanding how climatic and environmental factors shape ancient tree distribution is essential for their long-term conservation (Zhang et al., 2023; Xie et al., 2024b). Climate-driven environmental shifts are altering phenological cycles—such as earlier springs and delayed autumns—disrupting terrestrial carbon and water cycles (Piao et al., 2019) and destabilizing species habitats (Woodward and Williams, 1987). Additionally, anthropogenic pressures and soil properties, particularly cation exchange capacity, may significantly influence the habitat suitability of large ancient trees (Wan et al., 2020).

Understanding current distribution patterns and predicting suitable habitats for threatened species is fundamental to formulating effective conservation strategies (Tesfamariam et al., 2022; Wei et al., 2024). Species distribution models (SDMs) have emerged as powerful tools for evaluating environmental determinants and projecting range shifts under changing climatic conditions.

These modeling approaches can be broadly categorized into three types: profile-based methods (e.g., BIOCLIM, CLIMEX), regression-based methods [e.g., Generalized Linear Models (GLM), Generalized Additive Models (GAM)], and machine learning techniques [e.g., random forests, Maximum Entropy (MaxEnt) model, MaxNet, neural network models (NS)]. Commonly used Species Distribution Models (SDMs) include Generalized Additive Models (Hastie and Tibshirani, 1999), Generalized Linear Models (GLM) (Nelder and Wedderburn, 1972), random forests (Breiman, 2001), BIOCLIM (Busby, 1991), CLIMEX (Kriticos et al., 2015), Maximum Entropy (MaxEnt) model (Phillips et al., 2004), and MaxNet (Maxent over glmnet) model (Anderson and Handley, 2001).

The MaxEnt model has emerged as one of the most prevalent methodologies due to its predictive accuracy and model stability (Li et al., 2023), proving particularly effective for handling imbalanced and biased datasets (Ahmadi et al., 2022). Recent applications of MaxEnt in studying suitable habitats for ancient trees, endangered species, and notable woody specimens have yielded significant findings. For instance, environmental suitability assessments were conducted for the native Ethiopian tree species *Podocarpus falcatus* (Tesfamariam et al., 2022). Research on environmental drivers of endangered *Zelkova schneideriana* ancient trees in Hunan Province, China, identified solar radiation in May as a critical distribution determinant (He et al., 2024). MaxEnt modeling has also been utilized to analyze climate change impacts on the distribution of the rare and endangered Firmiana kwangsiensis (Gao et al., 2022), and to project future potential distributions of Pu’er ancient tea trees under 28 current environmental variables and future climate scenarios for sustainable management (Zhang et al., 2022). Additionally, MaxEnt applications under RCP 2.6 and RCP 8.5 climate scenarios have revealed potential distribution patterns for the endangered cervid subspecies Rucervus eldii eldii (Sangai) (Anand et al., 2021). China’s Second National Census of Ancient and Notable Trees (https://www.forestry.gov.cn/c/www/lczy/103688.jhtml) documented 5.0819 million specimens within the surveyed regions. Given the remarkable diversity of ancient and notable tree species, there remains an urgent need for extensive research to inform effective conservation management practices.

Among the ancient tree species, *Ficus altissima* Blume (*F. altissima*, subgenus Urostigma, section Conosycea), a monoecious hemi-epiphytic member of the Moraceae family (https://npgsweb.ars-grin.gov/gringlobal/search), holds a pivotal ecological position due to its exceptional vitality and adaptability. This keystone species thrives in diverse habitats ranging from mountainous regions to plains at elevations of 100–2000 m (Gargiullo, 2005), with its native distribution spanning the wet tropical biomes of Asia-Temperate and Asia-Tropical zones (Nguyen, 2006). Its range extends across multiple countries including China, Nepal, Bhutan, Malaysia, Thailand, and Indonesia, with significant populations in China’s Hainan, Guangxi, Yunnan, and Sichuan provinces (Zeng, 2013). *F. altissima* performs critical ecosystem services, including soil moisture regulation, air purification, and provision of essential foraging and nesting resources for frugivorous species (Chen et al., 2018; Debbarma et al., 2020). These ecological functions make it indispensable for maintaining regional biodiversity and ecological equilibrium. Beyond its ecological significance, ancient *F. altissima* specimens serve dual roles as both ornamental plants and sacred cultural symbols (Zhang et al., 2014). These venerable trees represent living fossils of the *Ficus* genus, embodying rich historical narratives and serving as tangible links to cultural heritage.

Contemporary research on *F. altissima* has predominantly explored its pharmacological applications (Hwisa et al., 2013; Yao et al., 2021, 2022; Chai et al., 2024), phylogeographic patterns (Huang et al., 2021), growth modulation by *Phoebe* species (Yao et al., 2022), genomic characterization (Chen et al., 2020; Zhang et al., 2021), nanomaterial development (Meng, 2015), and proteomic profiling (Dai et al., 2021). While these diverse research avenues highlight the species’ multifaceted significance, critical knowledge gaps persist regarding its climatic adaptability (Deng et al., 2023). Notably, systematic investigations into the responses of ancient *F. altissima* populations to contemporary and projected climate scenarios remain conspicuously absent from the literature.

The accelerating pace of climate change, compounded by intensifying anthropogenic pressures, is imposing unprecedented stress on natural ecosystems worldwide. These environmental perturbations are particularly consequential for Moraceae species, especially those within the ecologically vulnerable *Ficus* genus (Martínez-Macias et al., 2022). Climate models project a 0.1-meter rise in global mean sea levels by 2100, accompanied by temperature increases of 1.5–2°C under heightened anthropogenic forcing (IPCC, 2023). Such changes are amplifying the frequency and severity of extreme weather events - including prolonged droughts, catastrophic flooding, and lethal heatwaves - that collectively threaten *F. altissima* survival and reproduction. Parallel anthropogenic threats, notably urban encroachment, indiscriminate logging, and pervasive pollution, are synergistically degrading the species’ native habitats. This dual pressure system has already precipitated measurable shifts in the species’ distribution patterns and habitat suitability, potentially disrupting population dynamics and compromising long-term evolutionary potential.

Understanding the suitable habitats of ancient *F. altissima* trees represents a critical research priority that simultaneously advances ecological knowledge of the species’ environmental requirements and informs the development of science-based conservation frameworks. Such investigations provide essential insights into distributional patterns while establishing an empirical foundation for protecting this ecologically vital and culturally important species.

This study employs a combined approach of Geographic Information System (GIS) and Maximum Entropy (MaxEnt) modeling to achieve the following objectives: (1) reconstruct historical distribution patterns, (2) assess current habitat suitability, and (3) predict the future potential distribution range of the ancient *F. altissima* population in Guangxi Zhuang Autonomous Region, China, under different climate scenarios. The MaxEnt modeling method is based on Jaynes’s information theory principles (Jaynes, 1957), selecting the distribution with the highest entropy value under known constraints. It has advantages in handling complex nonlinear relationships and data with limited sample sizes (e.g., sparse distribution points of ancient trees) (Li et al., 2023). Through comprehensive habitat suitability modeling, this study will elucidate the species’ basic ecological niche requirements and generate scientific evidence to guide conservation planning and management decisions. We hypothesize that the spatial distribution of *F. altissima* is significantly dependent on three key environmental drivers: climatic parameters, soil characteristics, and topographic variables. By quantitatively analyzing these factors and their interactions, this study will generate high-resolution, spatially explicit predictions of suitable habitats. These findings will directly support the development of targeted conservation strategies for this key species and help maintain the integrity of associated ecosystems.

## 2 Materials and methods

### 2.1 Study area

**Figure 1:**
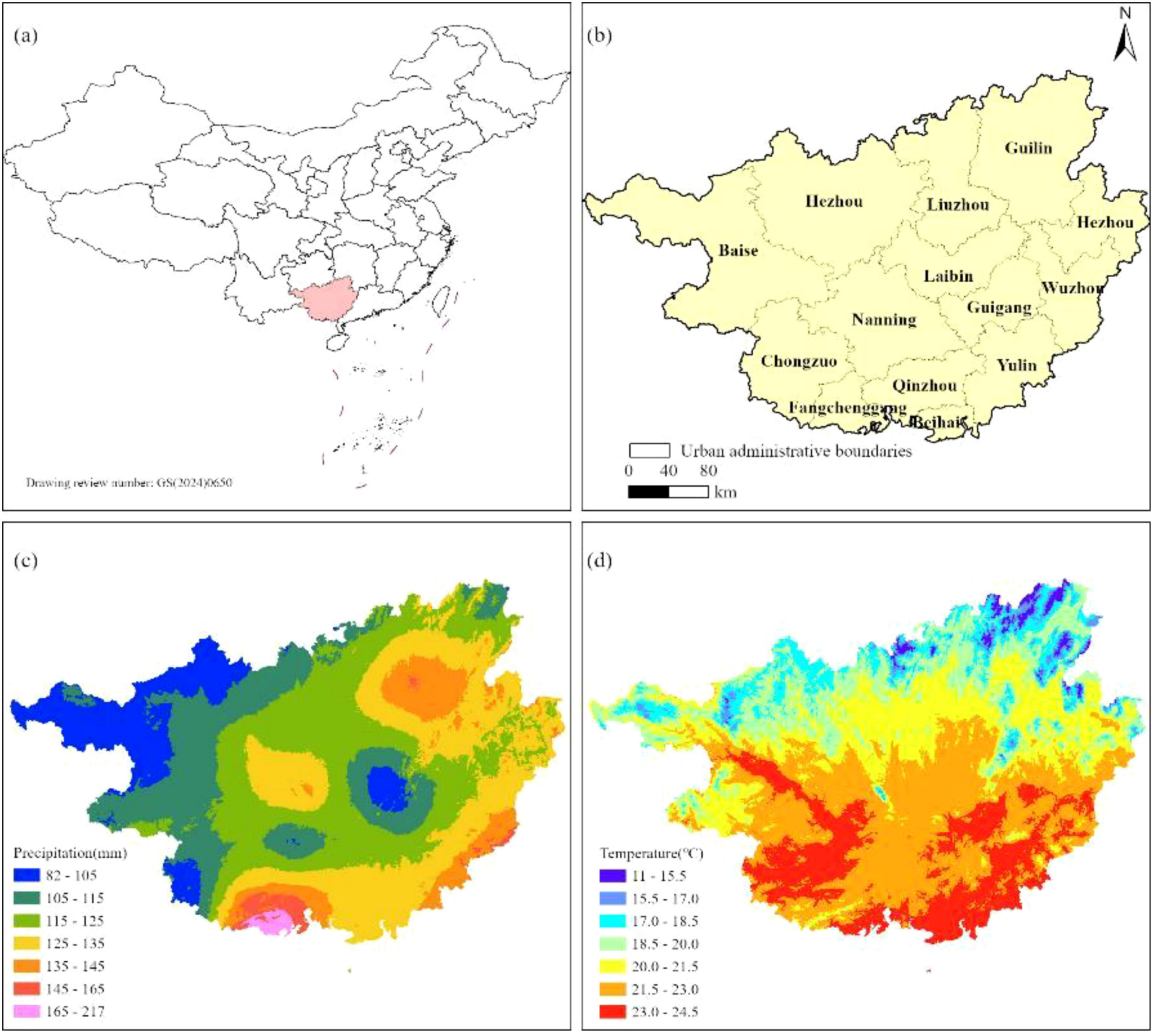
Overview of the study area.[**(a)** Guangxi's geographical location in China; **(b)** Administrative map of Guangxi; **(c)** Spatial distribution map of precipitation; **(d)** Spatial distribution map of temperature].

The Guangxi Zhuang Autonomous Region (104°26’–112°04’ E, 20°54’–26°24’ N) is situated along China’s southern coast (**Figure 1a**), adjacent to the Gulf of Tonkin and sharing a border with Vietnam to the southwest. Encompassing 237,600 km², the region is administratively organized into 14 prefecture-level cities (**Figure 1b**). Its topography exhibits a pronounced northwest-to-southeast gradient, featuring diverse landscapes that include mountain ranges, rolling hills, terraced plateaus, and alluvial plains. Climatically, Guangxi spans three distinct bioclimatic zones—northern tropical, southern subtropical, and mid-subtropical—with the Tropic of Cancer traversing its central region. The area is predominantly influenced by a subtropical monsoon climate, characterized by pronounced seasonal variations in temperature and precipitation. The northwestern Guangxi region exhibited significantly reduced precipitation levels (80–125 mm), contrasting sharply with the anomalously high rainfall observed in southern Guangxi (**Figure 1c**). Thermally, northern sectors of Guangxi registered below-average temperatures (11–15.5°C), while pronounced positive temperature anomalies dominated central and southern sectors, particularly encompassing the urban agglomerations of Nanning, Baise, Chongzuo, Guigang, and Yulin (**Figure 1d**). This unique geographic and climatic configuration supports rich biodiversity and complex ecosystems, making it an ecologically significant region in southern China.

The region’s exceptional biodiversity stems from its complex topographic features, abundant solar and thermal resources, optimal hydrothermal conditions, and varied soil composition. These environmental factors collectively foster a highly diverse flora with complex community structures, supporting a remarkable assemblage of ancient and valuable tree species. According to official registry data, Guangxi currently documents 156,512 protected ancient and valuable trees, comprising 156,371 ancient specimens - including 20,231 individuals from the ecologically significant Moraceae family. As a multiethnic autonomous region, Guangxi exhibits rich cultural diversity with populations of Han, Zhuang, Yao, Miao, Dong, Mulam, Maonan, Hui, Jing, Yi, Sui, and Gelao ethnic groups coexisting in the area. This unique combination of biological and cultural diversity makes the region particularly valuable for both ecological conservation and ethnobotanical studies.

### 2.2 Methodology

#### 2.2.1 Overview of the maximum entropy model

This study adopts an integrated modeling approach utilizing MaxEnt 3.4.4 and ArcGIS 10.8 to simulate and predict potential suitable habitats for ancient *F. altissima* populations across Guangxi. Our analytical framework incorporates a comprehensive dataset comprising 514 precisely georeferenced occurrence records and 33 multidimensional environmental variables spanning climatic, edaphic, and topographic parameters. Through rigorous variable contribution analysis and habitat suitability modeling, we aim to: (1) identify the key ecological determinants governing the species’ distribution, (2) delineate high-priority conservation areas, and (3) establish an evidence-based foundation for developing targeted management strategies to protect this ecologically keystone species.

#### 2.2.2 Model parameter configuration and execution

In order to significantly improve predictions, a random method was employed to thin out the ancient tree records geographically based on spatial autocorrelation. Model parameter settings include the random test percentage, maximum iterations, response curves, Receiver Operating Characteristic (ROC) curves, and the Jackknife test. During model execution, the random test percentage was set to 25%, meaning 75% of the occurrence points were used for model training and 25% for validation to ensure model generalizability. The maximum number of iterations was fixed at 10 to guarantee convergence to an optimal solution. ROC curves and response curves were employed to evaluate model accuracy and determine the suitability ranges of ecological variables for *F. altissima* distribution. Response curve analysis identified the optimal and marginal suitability thresholds for each variable. Model prediction accuracy was assessed using the Area Under the ROC Curve (AUC), where higher AUC values indicate superior predictive performance (Fielding and Bell, 1997; Wu et al., 2007). The Jackknife test (Muyobela et al., 2023) was applied to evaluate the relative importance of ecological variables, with Jackknife plots revealing the dominant drivers of species distribution. Continuous parameter tuning ensured high precision and reliability in model outputs.

The MaxEnt modeling framework primarily involves environmental variable selection, modeling procedures, and parameter optimization. Multicollinearity among environmental variables can distort response relationships and contribution rates, thereby compromising model accuracy (Graham, 2003; Zenebe et al., 2024). Consequently, variable selection is critical for simulation fidelity, necessitating Pearson correlation analysis to eliminate highly correlated factors and enhance prediction robustness.

#### 2.2.3 Biomass estimation methods

The aboveground biomass of each *F. altissima* ancient tree was estimated using the BIOMASS package (Réjou-Méchain et al., 2017). To account for uncertainty, the Monte Carlo method was applied with 1,000 iterations to simulate stand-level aboveground biomass for individual trees (Chave et al., 2004).

#### 2.2.4 Future scenario settings

SSP (Shared Socioeconomic Pathways), which combines socioeconomic assumptions and radiative forcing levels, was used to predict future climate change (Riahi et al., 2017). To investigate the suitability distribution of *F. altissima* under future climate scenarios, we analyzed four periods (2021–2040, 2041–2060, 2061–2080, and 2081–2100) based on the highest (SSP5-8.5) and lowest (SSP1-2.6) emission scenarios. Model accuracy evaluation results (**Supplementary Figures S6–S13**) showed that all model accuracies were above 0.90.

### 2.3 Data sources

The sample data were primarily collected by accessing the Guangxi Ancient and Valuable Trees Management System (https://www.gxoldtree.com.cn/) to gather information on ancient *F. altissima* trees aged 300 years or older in the region, including the precise geographical locations and growth status data of 616 individual trees. Latitude and longitude coordinates of each specimen were verified using Google Earth (http://ditu.google.cn). Spatial filtering was applied to the ancient tree occurrence data using ENMTools (Warren et al., 2010) to remove redundant records (e.g., anthropogenic spatial clustering around villages), thereby minimizing potential sampling bias. This process yielded a final dataset of 514 unique georeferenced occurrences. One-way analysis of variance (ANOVA) and Duncan’s multiple range test were applied to assess significant differences (P< 0.05) in tree height, diameter at breast height (DBH), crown width, and species typology. Additionally, distribution data were supplemented and refined through comprehensive reviews of relevant scientific literature and technical reports to ensure the completeness and representativeness of the dataset.

The foundational geospatial data were derived from the national standard vector maps provided by the National Geospatial Information Public Service Platform (https://www.tianditu.gov.cn/). Environmental data encompassed multi-dimensional information on climate, soil, and topography: (1) bio1-19 data were obtained from the WorldClim database (https://worldclim.org) at 30 arc-second resolution (**Supplementary Table S1**). (2) Soil variables were derived from the Soil Grids database (https://www.soilgrids.org) with 1×1 kilometer resolution. ArcGIS 10.8 was employed to resample parameters to 30 arc-second spatial resolution (**Supplementary Table S2**). (3) Digital Elevation Model (DEM) data were sourced from the China Geospatial Data Cloud Platform (https://www.gscloud.cn/home) using 90m-resolution ASTER GDEM data.

By integrating these datasets, a comprehensive analysis of the habitat conditions for ancient *F. altissima* trees was conducted, providing robust data support for the implementation of the Maximum Entropy (MaxEnt) model. To assess potential sampling point bias, we analyzed the distribution of ancient trees relative to roadsides and nature reserves. Specifically, we utilized Guangxi Zhuang Autonomous Region road data (https://www.openstreetmap.org/) to create 20-meter buffer zones on both sides of roads. Analysis revealed that only 32 ancient trees fall within these roadside buffers. Similarly, only 9 ancient trees are located within nature reserves (**Supplementary Figure S1**). These findings collectively indicate minimal bias in our ancient tree sampling points.

### 2.4 Technical workflow

**Figure 2:**
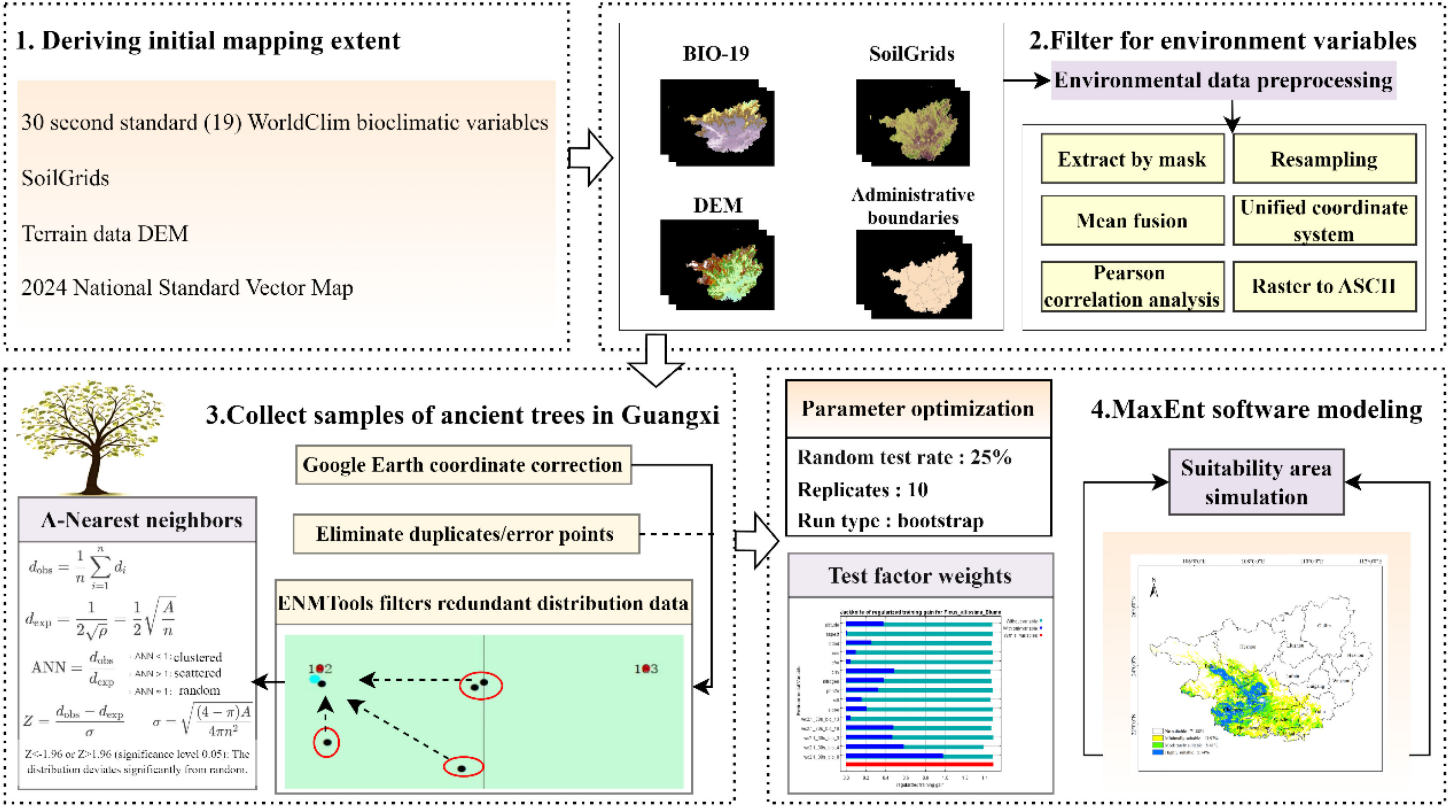
Research technical workflow diagram.

Based on the methodologies and data sources described in the preceding sections, a technical workflow diagram illustrating the framework of this study was developed (**Figure 2**).

## 3 Results and analysis

### 3.1 Analysis of ancient *F. altissima* tree characteristics

**Figure 3:**
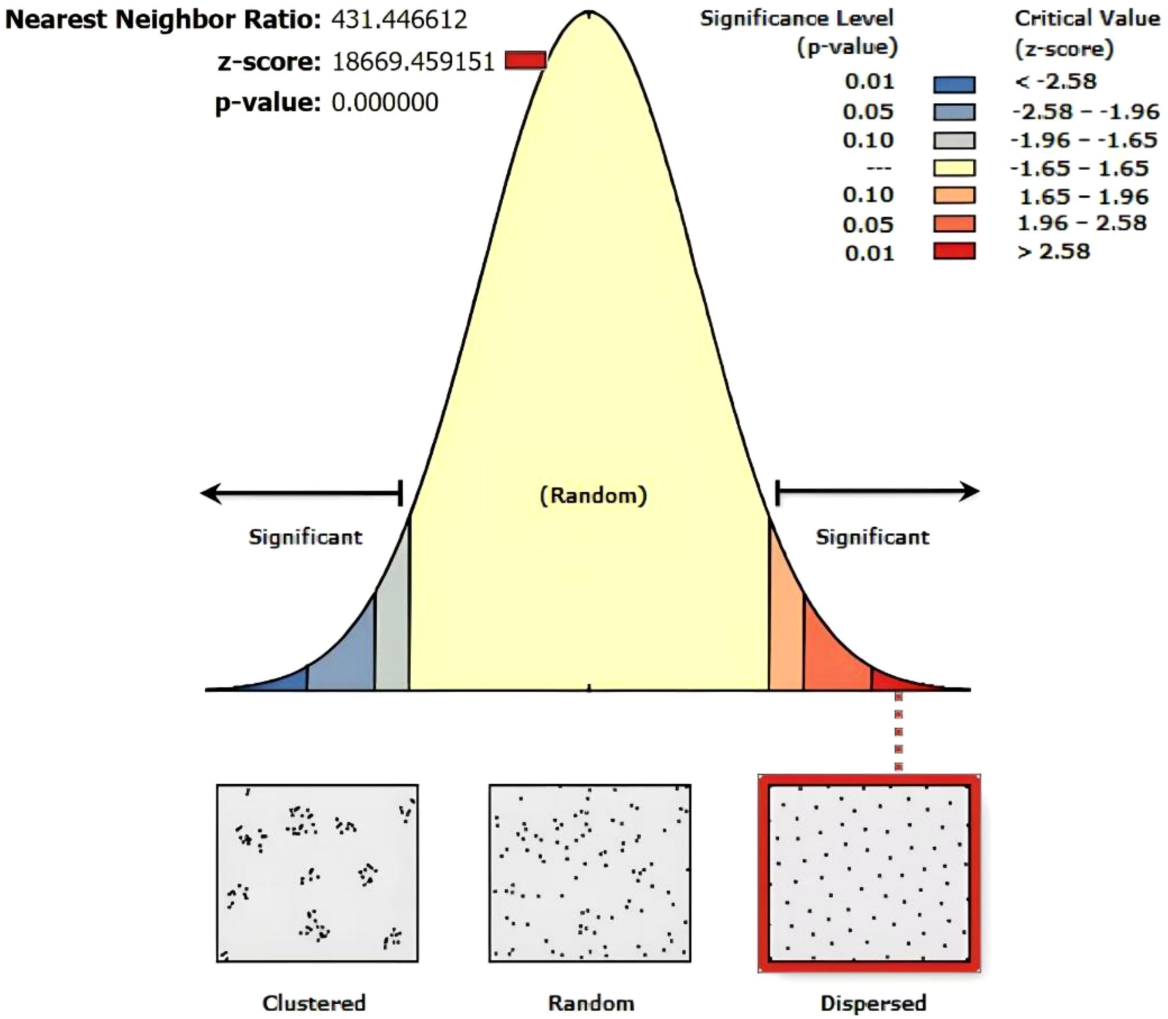
Nearest neighbor analysis of *F. altissima* ancient trees distribution.

This study investigated the spatial distribution, age, size (height, diameter at breast height (DBH), crown width), and growth status (habitat conditions and vitality) of ancient *F. altissima* trees. A total of 110 first-grade ancient trees (aged ≥500 years) were identified, including one specimen exceeding 1,000 years. Additionally, 404 second-grade ancient trees (aged ≥300 years) were documented. Among these, 13 trees are classified as endangered, while the remaining 101 exhibit weakened growth vigor. The tree age of ancient Ficus altissima exhibited a significant correlation with DBH, with a correlation coefficient of 0.64. Tree height was significantly correlated with mean crown width (**Supplementary Figure S2**). In the Average Nearest Neighbor (ANN) analysis (**Figure 3**), the spatial distribution of ancient *F. altissima* trees were significantly dispersed, as indicated by a p-value<0.001 and a z-score >2.58.

**Figure 4:**
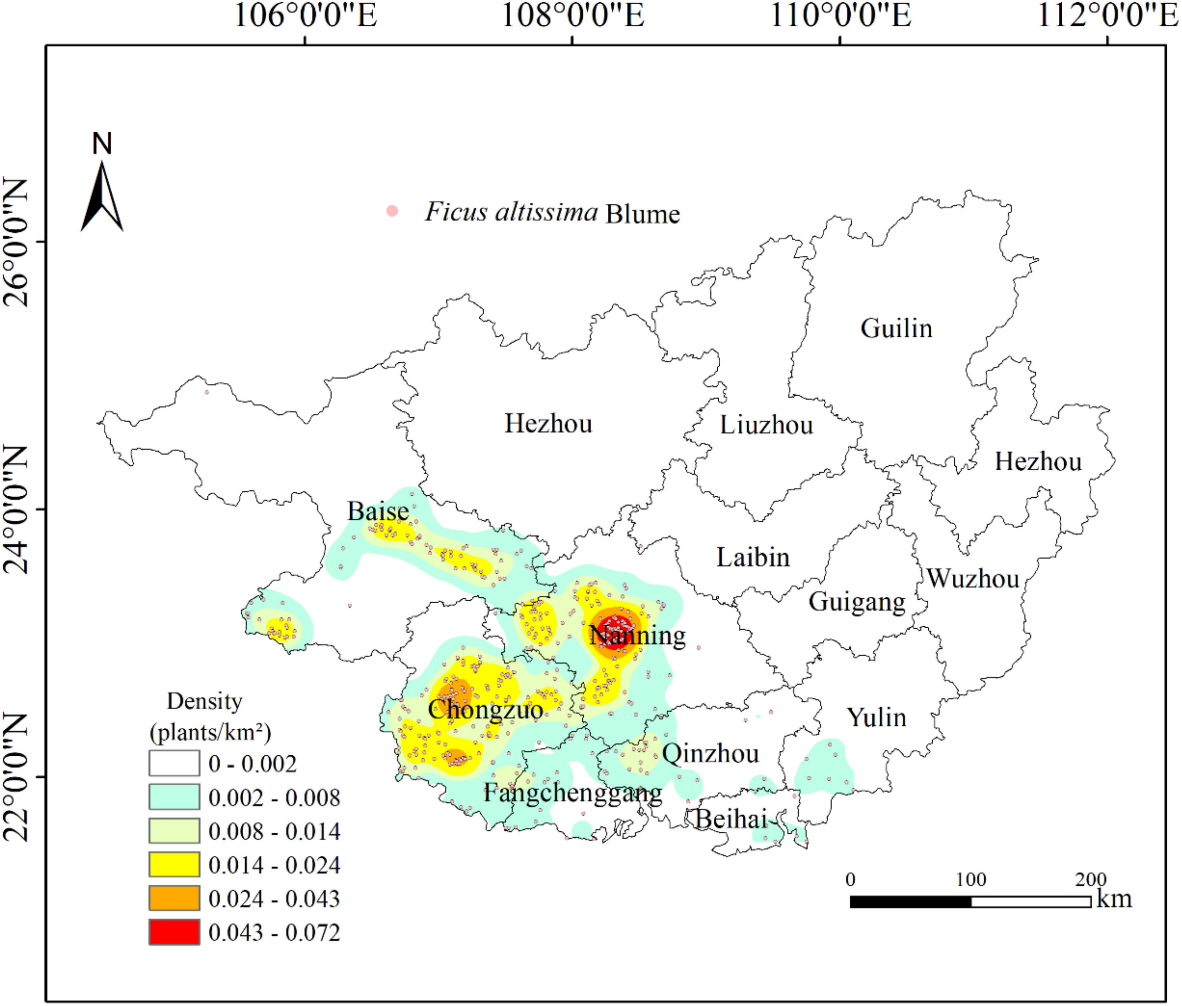
Kernel density analysis.

The Kernel Density Analysis results revealed three significant spatial clusters of ancient *F. altissima* trees within the Guangxi Zhuang Autonomous Region (**Figure 4**). One high-density cluster was identified in Nanning City, with a density range of 0.043–0.072 individuals/km², while two sub-density clusters were observed in Chongzuo City, exhibiting a density range of 0.024–0.043 individuals/km². Nanning and Chongzuo were identified as the core distribution areas for ancient *F. altissima* trees, where the local mean elevation ranges from 100 to 300 meters.

**Figure 5:**
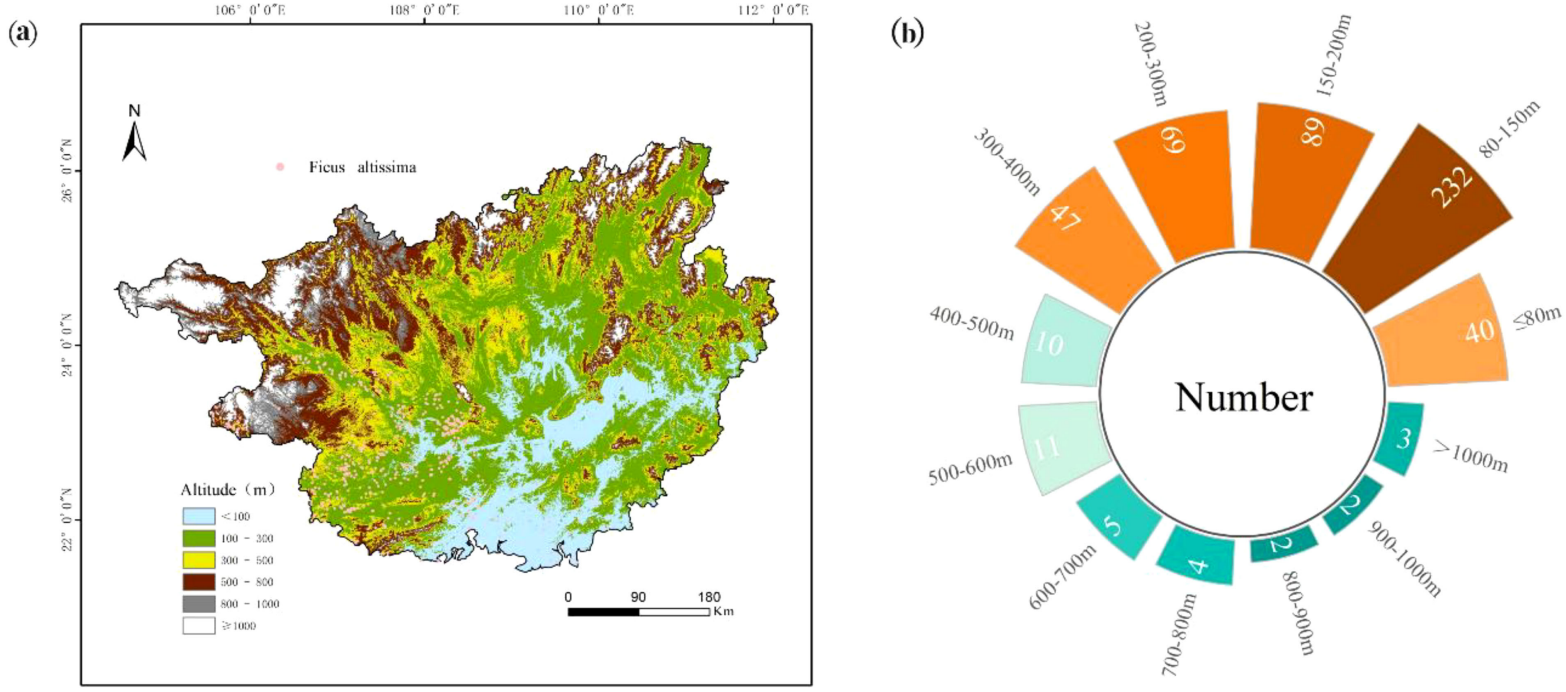
Topographic distribution of *F. altissima* ancient trees in Guangxi Province [**(a)** Elevation profile; **(b)** Elevation zone statistics].

Analysis of ancient *F. altissima* distribution revealed distinct elevational patterns. The species predominantly clustered in mid-low altitude regions (80–400 m asl), with the highest density recorded at 80–150 m (232 individuals), followed by 150–200 m (89 individuals). Secondary aggregations occurred at higher elevations: 200–300 m asl and 300–400 m asl. Below 80 m asl—characterized by flat terrain and poor soil drainage—distribution density significantly declined, indicating suboptimal habitat conditions. Conversely, above 600 m asl, population numbers progressively decreased with increasing elevation. These distribution patterns are further validated by the elevational distribution profile (**Figure 5a**) and radial bar chart (**Figure 5b**).

**Figure 6:**
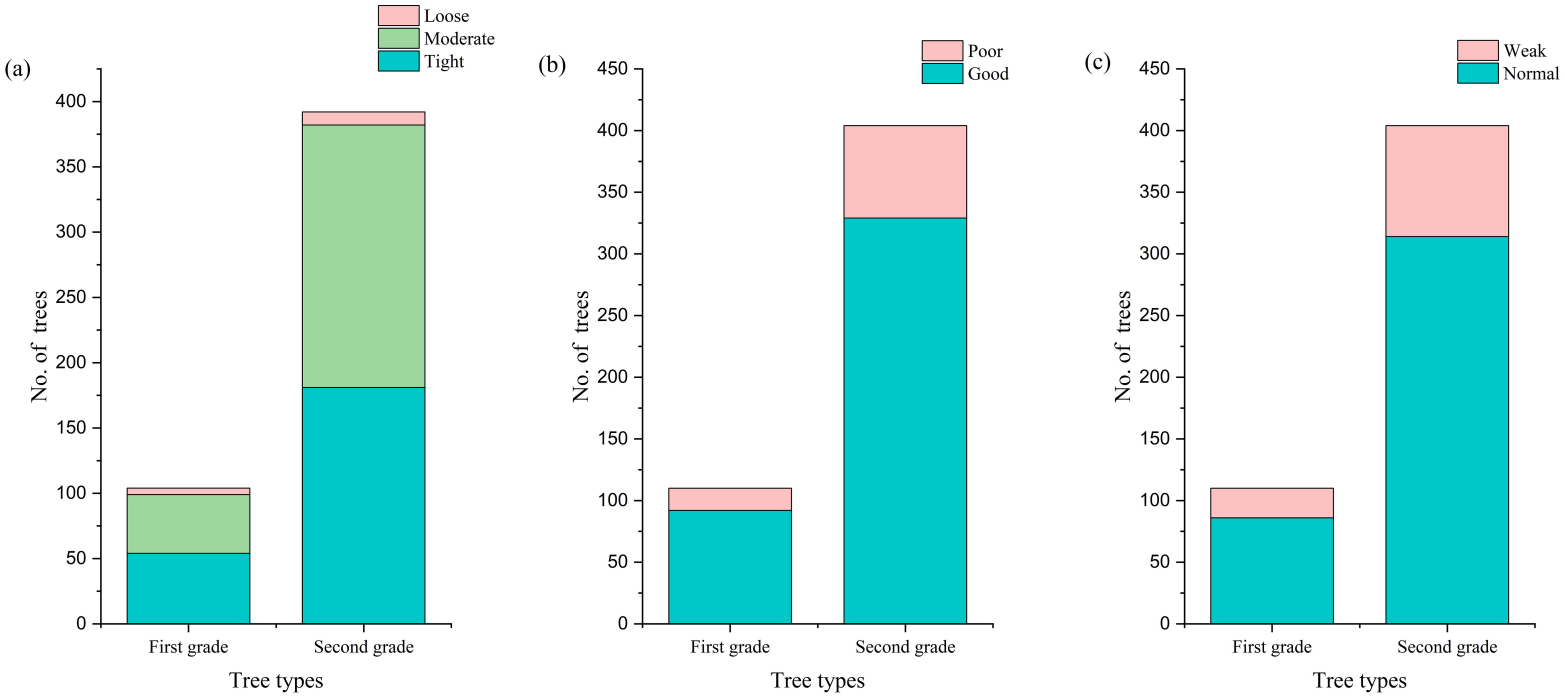
Comparison of growth environments for grade I and grade II ancient trees [**(a)** Soil compactness; **(b)** Environmental quality; **(c)** Growth vigor].

Comparing the growth environment conditions and growth status of first- and second-grade *F. altissima* ancient trees, we found that both grades of ancient trees grow in relatively compact soil, but with slight differences: the highest proportion of first-class ancient trees grow in compact soil, while second-class ancient trees have the largest proportion in moderately compact soil (**Figure 6a**). However, excessively compact soil environments often lead to soil compaction issues, causing multiple adverse effects on ancient trees: reducing soil permeability, restricting root respiration, and thereby affecting normal growth and causing decline. It also limits root expansion, potentially leading to massive root death, which seriously impacts tree stability and water/nutrient absorption capacity. Compact soil may also result in nutrient deficiencies, causing slow growth, sparse foliage, and weakened stress resistance. Therefore, to promote healthy growth of ancient trees, measures should be implemented to improve soil conditions, such as regular topsoil loosening to enhance air exchange, and burying branches/leaves around the outer canopy perimeter to increase soil porosity. Additionally, even ancient trees growing in favorable environments still face the risk of decline and endangerment, as shown in the comparison between **Figures 6b, c**.

**Figure 7:**
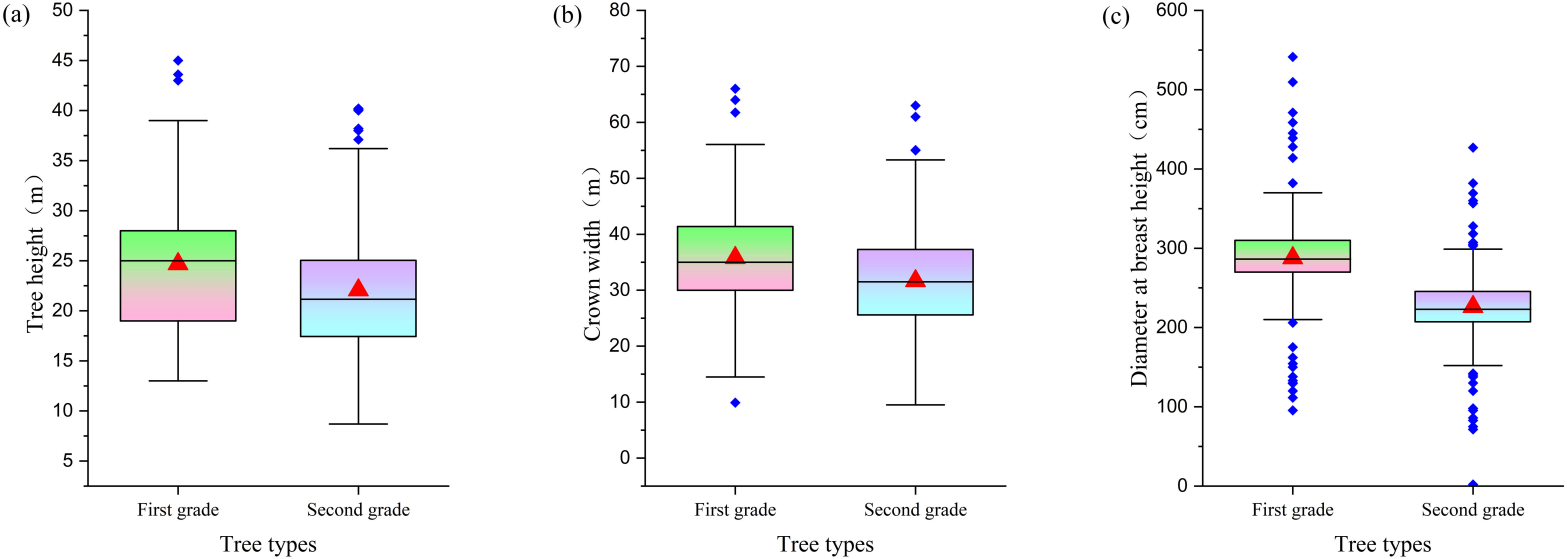
Comparison of morphological characteristics between grade I and grade II ancient trees [**(a)** Tree height distribution; **(b)** Crown width distribution; **(c)** DBH distribution].

Research has confirmed that there are significant differences in growth morphology between first- and second-class ancient trees. In terms of average tree height, first-class ancient trees are typically 5–8 meters taller than second-class ancient trees (**Figure 7a**). A similar trend is observed in average crown spread, with first-class ancient trees having an average crown spread that is 5–6 meters larger than that of second-class ancient trees (**Figure 7b**). The significant differences between the two levels of ancient trees are further highlighted by their average DBH: the average DBH of first-level ancient trees is approximately 50 centimeters larger than that of second-level ancient trees, underscoring the significant structural differences between the two (**Figure 7c**).

**Figure 8:**
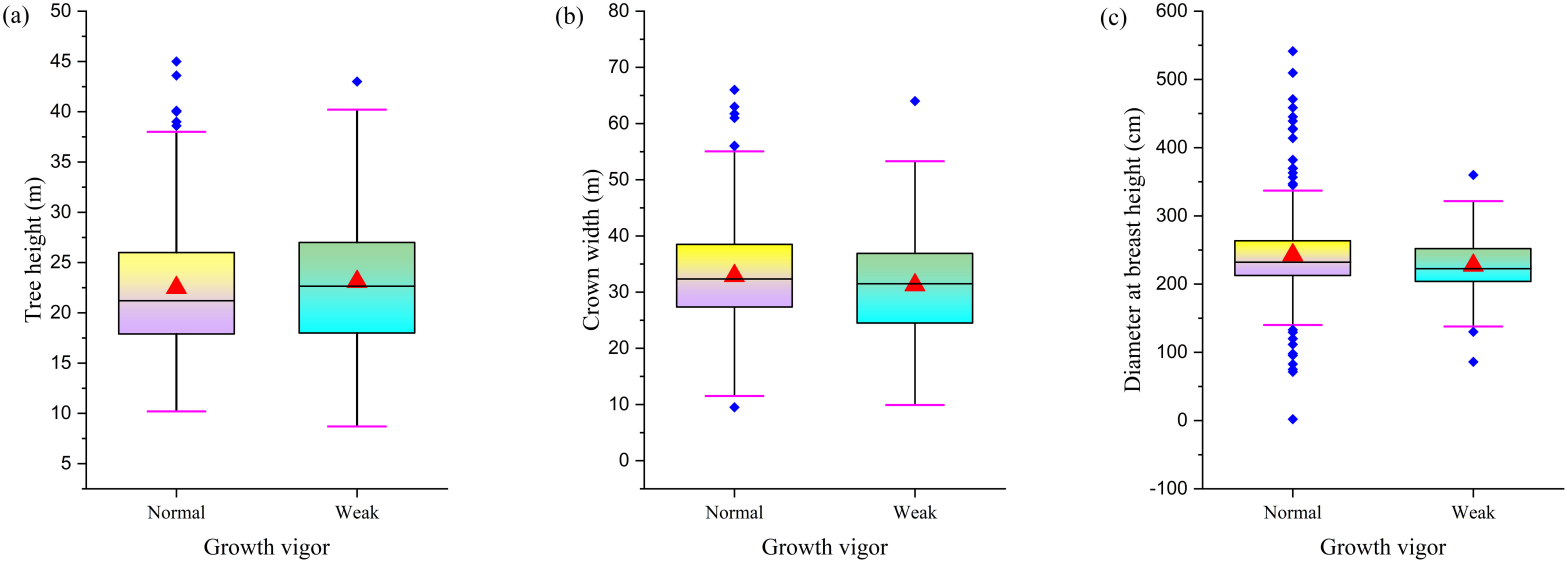
Comparison of morphological characteristics between differential growth vigor trees. [**(a)** Tree Height Distribution; **(b)** Crown Width Distribution; **(c)** DBH distribution].

A comparative study of *F. altissima* ancient trees with different growth vigor revealed significant differences in growth indicators between weakened and healthy individuals. Although the average tree height of weakened individuals was approximately 2.5 meters taller than that of healthy individuals (**Figure 8a**), the average crown spread of healthy individuals was 5–6 meters larger than that of weakened individuals (**Figure 8b**), and the average diameter at breast height of healthy individuals was approximately 50 centimeters larger than that of weakened individuals (**Figure 8c**). These data reveal the association between growth vigor and tree structure. Although weakened individuals have a slight advantage in height, healthy individuals exhibit superior performance in crown spread and trunk diameter development.

**Figure 9:**
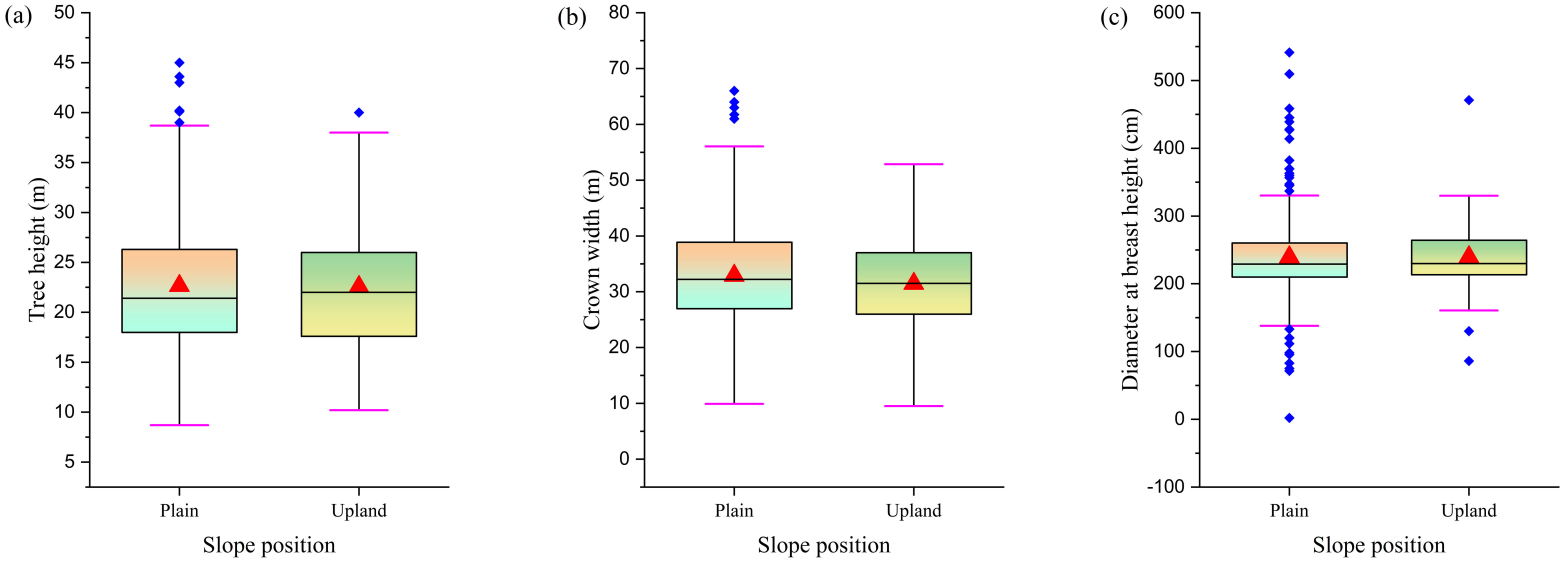
Comparison of morphological characteristics in ancient trees across different slope positions [**(a)** Tree height distribution; **(b)** Crown width distribution; **(c)** DBH distribution].

Research has found that *F. altissima* ancient trees growing on flat terrain and slopes exhibit minimal differences in average tree height (**Figure 9a**) and average breast height diameter (**Figure 9b**), but show significant differences in average crown width (**Figure 9c**). The crown width of ancient trees on plain is, on average, 5 meters larger than that of ancient trees on upland. This difference is primarily attributed to the relatively uniform terrain and climate conditions in flat areas, which provide stable environmental factors such as suitable temperature, sunlight, and moisture, thereby creating a stable environment for tree growth, allowing trees to fully develop and expand their crown width. Additionally, flat terrain typically has uniform soil conditions, providing plants with ample nutrients and spatial resources for growth. In contrast, mountainous or sloping terrain, with its complex topography and variable climate conditions, imposes greater constraints and challenges on plant growth, resulting in relatively smaller crown sizes. The relevant conclusions are supported by the data analysis in **Figure 9**, where the noticeable gap in crown size in **Figure 9b** visually demonstrates the influence of terrain on the development of ancient tree crown size.

### 3.2 Relationship between tree age and biomass

**Figure 10:**
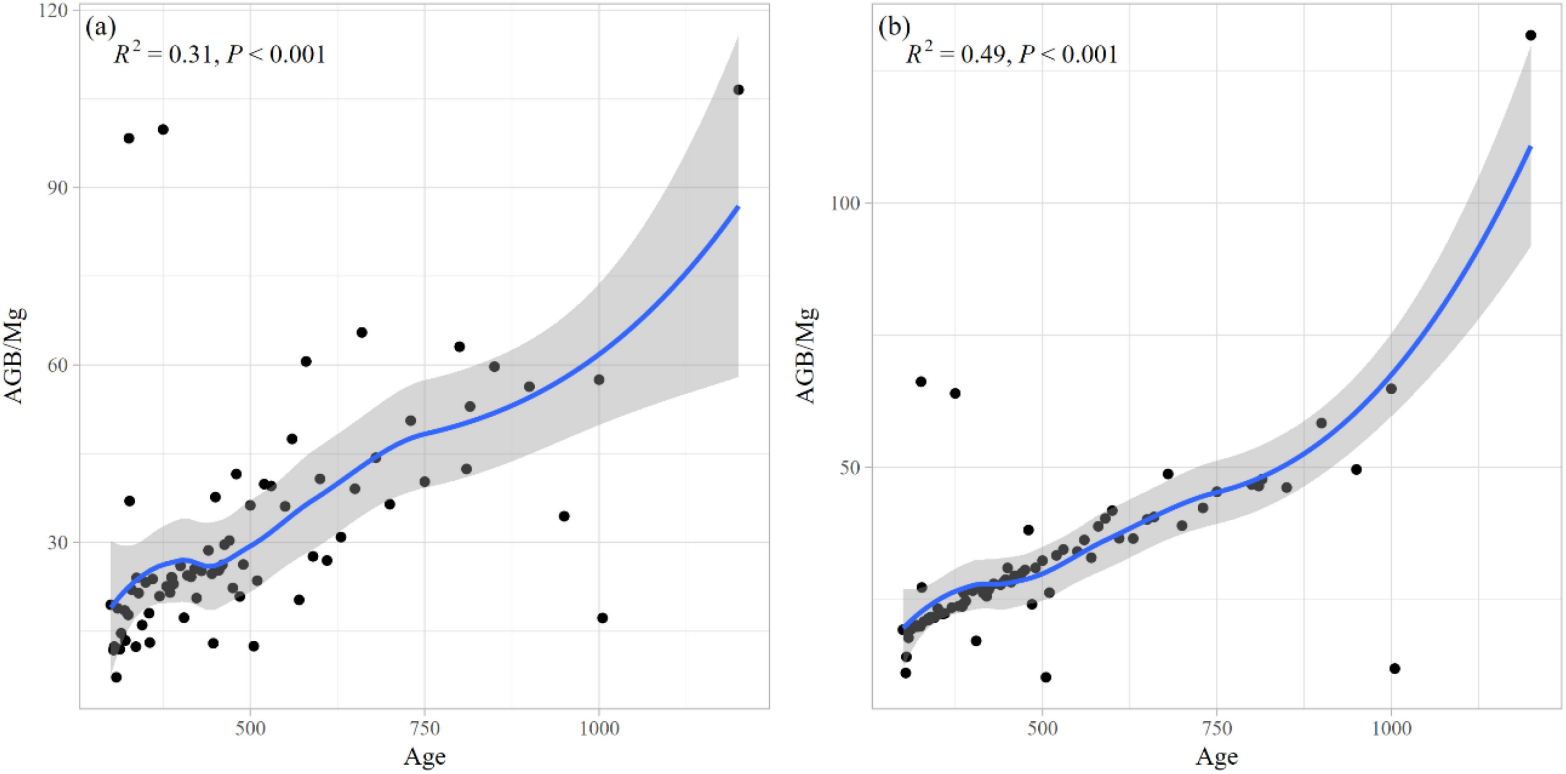
The above ground biomass (AGB) of *F*. *altissima* ancient tree **(a)**, and the AGB at stand level with the error associated to the wood density **(b)**.

The mean biomass values were calculated for each age group, and the results are presented in **Figure 10**. The analysis revealed a statistically significant correlation between tree age and biomass for both estimation methods, with determination coefficients (*R*²) of 0.31 (**Figure 10a**) and 0.49 (**Figure 10b**), respectively. The total aboveground biomass of all sampled ancient *F. altissima* trees was approximately 2,388.833 Mg, while the mean stand-level aboveground biomass was 2,410.085 Mg, indicating close agreement between the two measures.

### 3.3 Model accuracy evaluation

**Figure 11:**
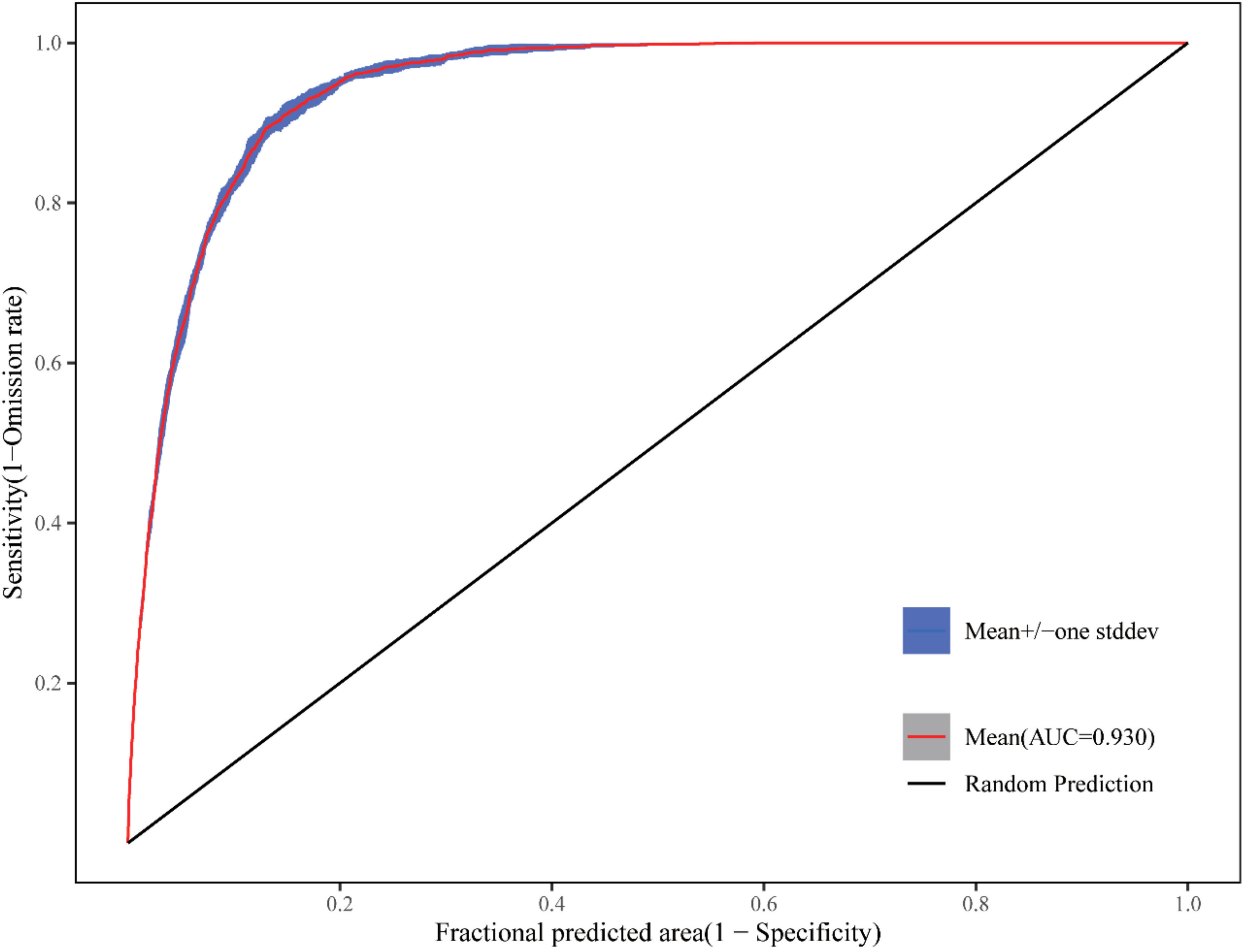
ROC curve of MaxEnt model prediction.

In this study, the model was run 10 times with averaged results, generating the ROC (Receiver Operating Characteristic) curve (**Figure 11**). The mean training AUC value was 0.93 with a standard deviation of ±0.002, demonstrating high stability across model replicates. According to evaluation criteria, the model achieved excellent accuracy in predicting the suitable habitat distribution of *F. altissima* ancient trees, indicating its robust capability to simulate their potential geographic range.

**Figure 12:**
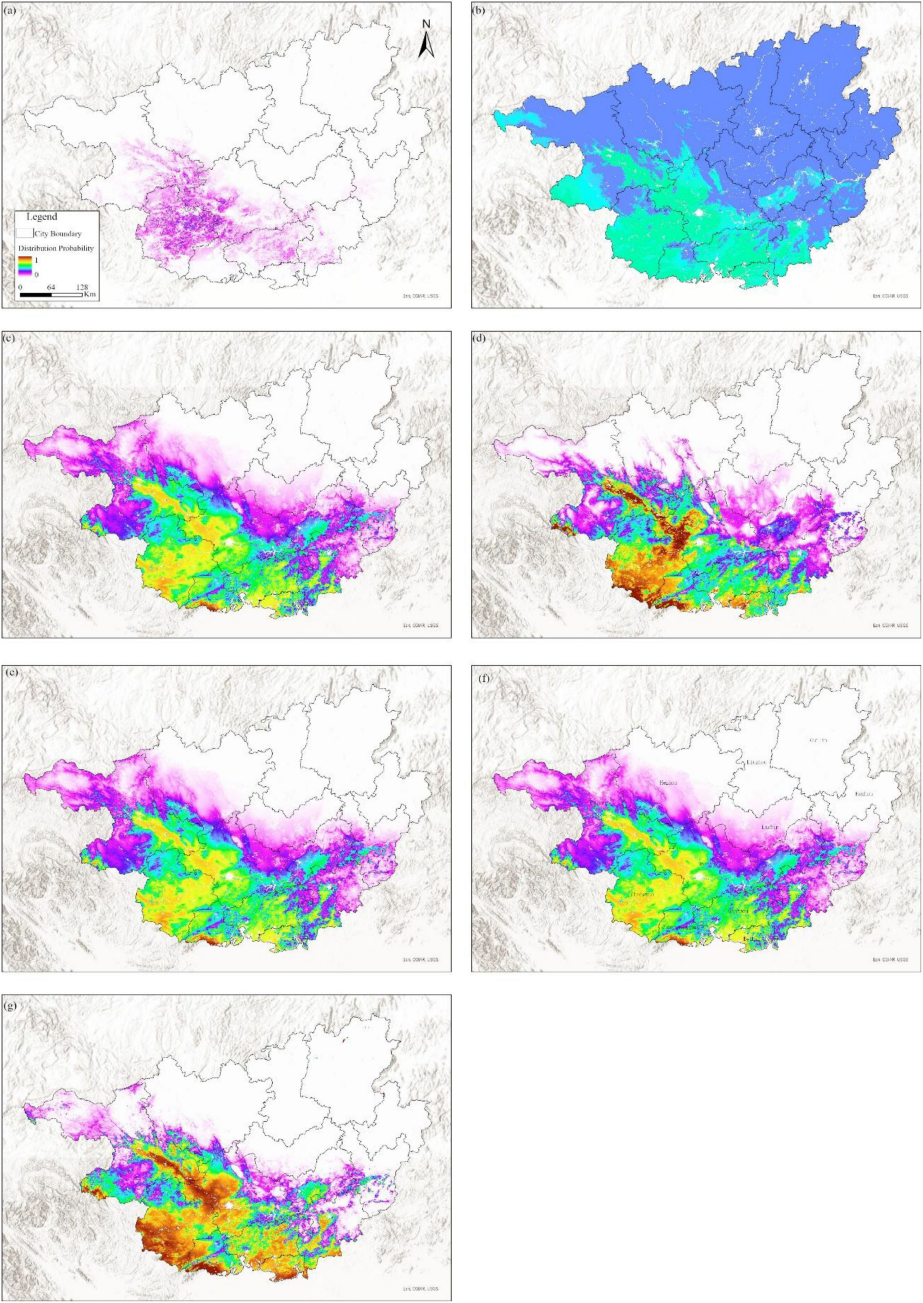
Comparison of simulation results from various models based on current environmental and climatic factors [**(a)** BIOCLIM, **(b)** BRT, **(c)** GAM, **(d)** GLM, **(e)** MaxEnt, **(f)** MaxNet, **(g)** NS].

To validate the applicability of the MaxEnt model for *F. altissima* ancient trees in Guangxi, this study compared it with several widely used models, including the Bioclim model, MaxNet model, Generalized Linear Model (GLM), Generalized Additive Model (GAM), Natural Splines (NS), and Boosted Regression Tree (BRT). Based on the enmSdmX (Smith et al., 2023) and predicts (Hijmans, 2024) R language package, the simulation results of these models are shown in **Figure 12**, and their accuracy evaluations are summarized in **Supplementary Table S3**.

All SDM generally identified consistent core areas for the *F. altissima* ancient trees, with variation among models occurring outside of those core areas. In terms of model accuracy, the BIOCLIM model, BRT, GAM, MaxNet, and MaxEnt model achieved AUC values above 0.85. However, the MaxEnt model exhibited the highest correlation coefficient (0.73). Regarding the spatial characteristics of the simulated suitable habitats for *F. altissima* ancient trees, the distribution ranges predicted by the MaxEnt model closely aligned with those of other models (except for the Random Forest model), demonstrating strong consistency in spatial patterns.

### 3.4 Analysis of environmental factors in suitable habitats

**Figure 13:**
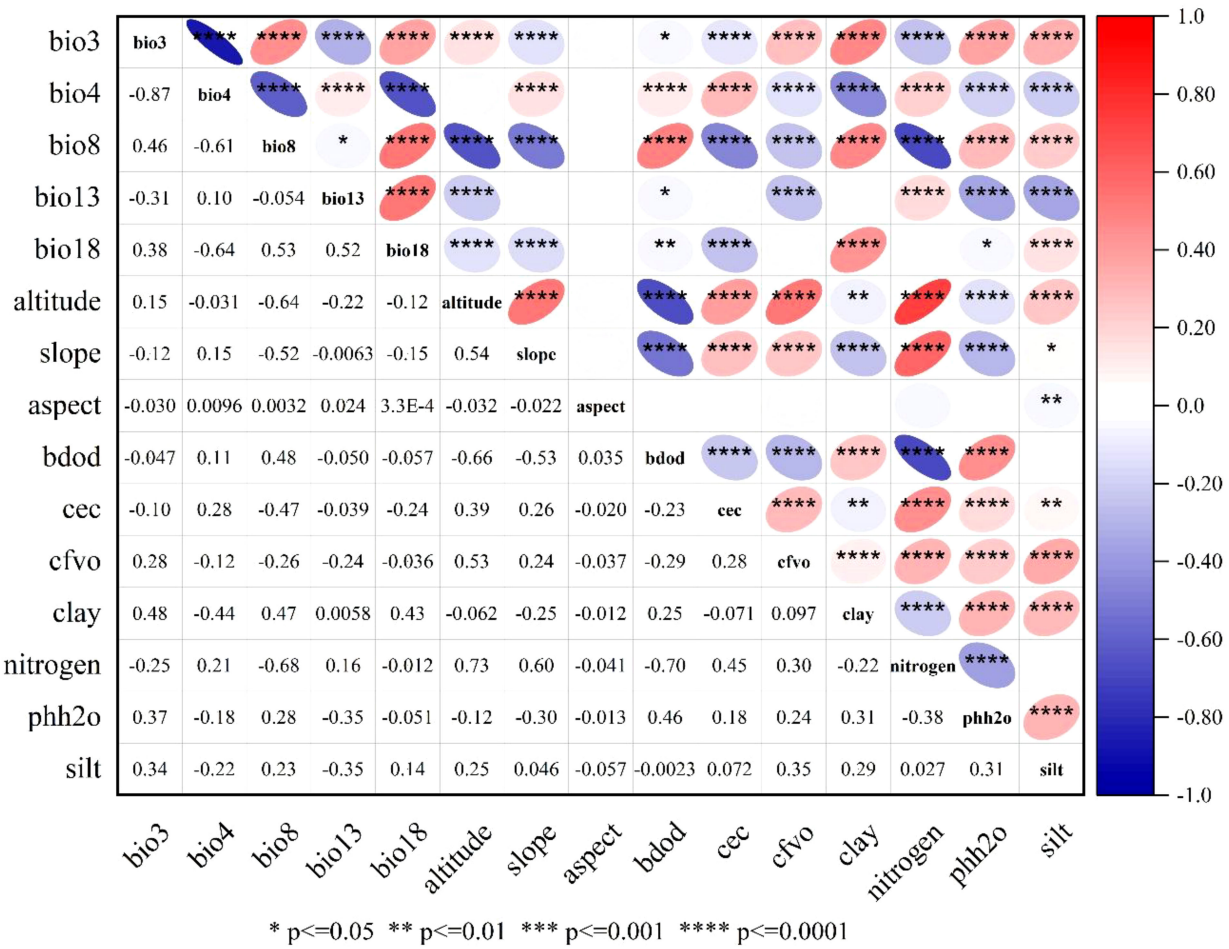
Heatmap of climatic-environmental factor correlation analysis.

Further analysis of environmental factors within suitable habitats identified climatic conditions and soil types as key determinants of *F. altissima* distribution (**Figure 13**). Notably, optimal growth occurred in regions with higher mean temperatures during the wettest quarter, where elevated temperatures enhance surface evapotranspiration and plant transpiration, thereby supporting physiological activity in humid seasons (Zheng et al., 2024). Additionally, temperature seasonality, soil gravel content, and topographic elevation significantly influenced the species’ spatial distribution patterns.

Specifically, *F. altissima* thrives in warm and humid climates, with optimal growth observed in regions where the mean annual temperature ranges between 20–25°C and annual precipitation falls within 1,500–2,000 mm. Regarding soil types, ancient *F. altissima* trees predominantly occur in lateritic red soils and mountainous red soils, which are characterized by high fertility, good drainage, and a pH range of 4.5–6.5—conditions highly conducive to their growth. Soil organic matter (SOM) content is another critical factor, as soils with elevated SOM levels provide abundant nutrients that enhance root development and biomass accumulation.

In terms of topography, moderately sloped mountainous areas (slope gradients: 10–30°) are most favorable for ancient *F. altissima* growth. Excessively steep (>30°) or gentle (<10°) slopes impede root anchorage and water retention. Furthermore, northeast-facing slopes and southeast-facing slopes exhibit optimal light exposure and humidity conditions, facilitating photosynthesis and water uptake—key physiological drivers of growth in humid subtropical ecosystems.

### 3.5 Jackknife test for variable contribution scores

MaxEnt analysis quantified the percentage contributions of 15 variables to the habitat suitability modeling of ancient *F. altissima* (**Table 1**). Two climatic factors—mean temperature of the wettest quarter (bio8) and temperature seasonality (bio4)—emerged as dominant predictors, each contributing >15% to the model. Secondary contributors included clay fraction in fine-grained soils and elevation. Although variables such as mean precipitation of the warmest quarter (bio18), ratio of diurnal to annual temperature range (bio2), and total nitrogen content exhibited lower overall contributions, their irreplaceability in the model was confirmed, as they captured unique ecological gradients not replicated by other predictors.

**Table 1:** Importance of individual climatic variables in the MaxEnt model.

Environment factor	Description	Contribution rate (%)	Importance of replacement (%)
bio8	Mean temperature of wettest quarter	59.1	7.6
bio4	Temperature seasonality	18.5	49.3
clay	Proportion of clay particles in the fine earth fraction	4.7	5.9
altitude	DEM	4.3	4
phh2o	Soil pH	2.3	0.8
nitrogen	Total nitrogen (N)	1.9	6.9
silt	Proporltion of silt particles in the fine earth fraction	1.8	4.1
bio3	Isothermality	1.8	0.9
bio18	Precipitation of warmest quarter	1.4	12.1
bdod	Bulk density of the fine earth fraction	1	3.3
cfvo	Volumetric fraction of coarse fragments	0.8	0.8
bio13	Precipitation of wettest month	0.8	2.4
slope	Slope	0.7	1
aspect	Aspect	0.6	0.6
cec	Cation Exchange Capacity of the soil	0.3	0.3

**Figure 14:**
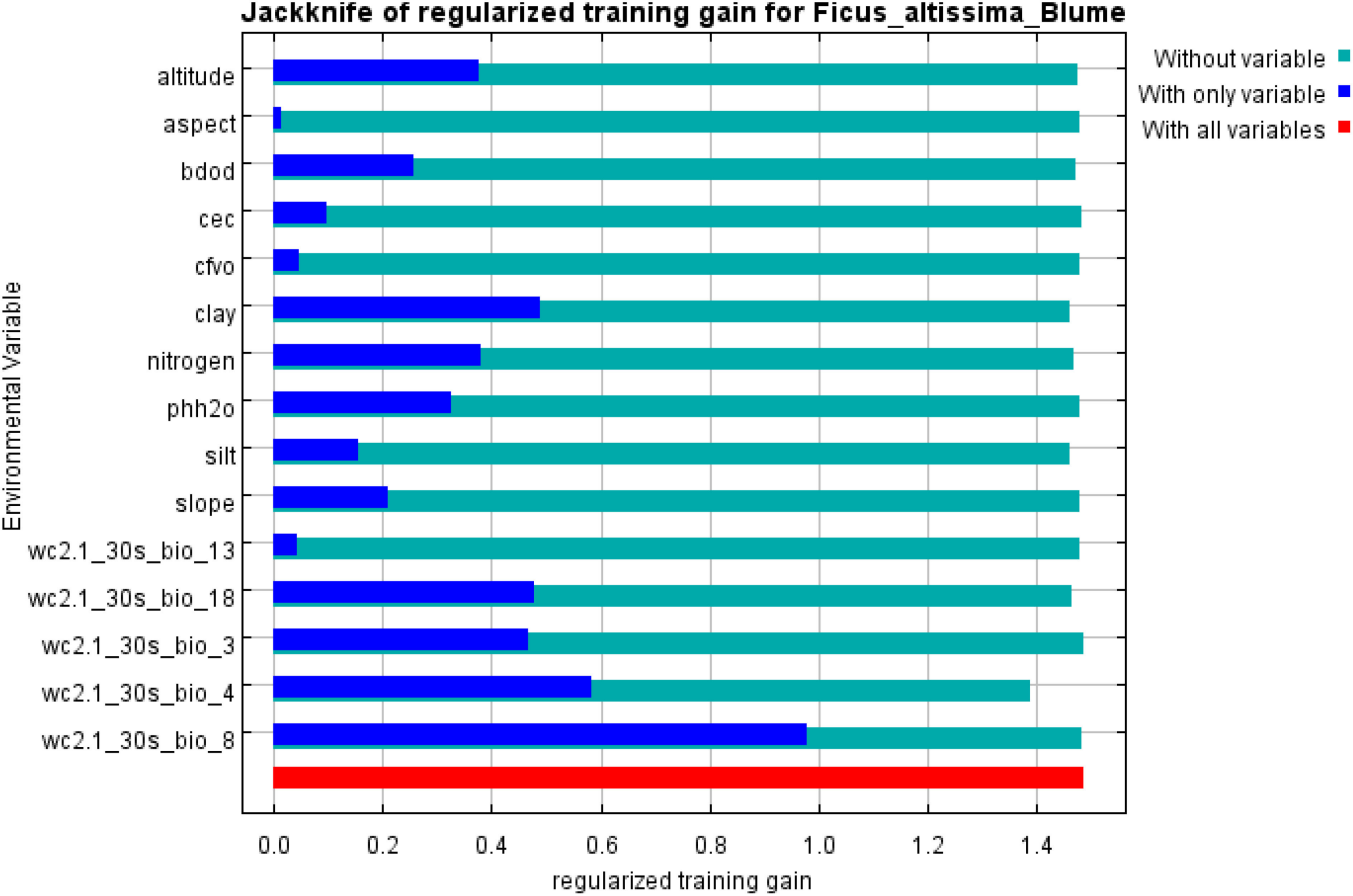
Jackknife test the importance of environmental variable.

A Jackknife analysis of environmental factors, based on the regularized training gain of the MaxEnt model (**Figure 14**), revealed that the four variables with the highest individual contributions to model accuracy were: mean temperature of the wettest quarter (bio8), temperature seasonality (bio4), clay fraction in fine-grained soils, and mean precipitation of the warmest quarter (bio18), all demonstrating strong predictive capacity for the distribution of ancient *F. altissima*. Notably, Aspect exhibited the shortest blue bar (lowest score when used alone), yet its exclusion significantly reduced model performance (green bar), indicating its unique topographic information (e.g., microclimate or solar radiation effects) remains essential for explaining the species’ distribution patterns.

### 3.6 Response analysis of habitat suitability and key variables for ancient *F. altissima* distribution

**Supplementary Figures S3–S5** display the response curves for three key variables (out of the 15). The response curve for the mean temperature of the wettest quarter (bio8) reveals that as temperature increases from 24°C, the habitat suitability index for ancient *F. altissima* rises sharply, reaching its peak and stabilizing when the temperature exceeds 29°C. This indicates a high thermal requirement for the species’ survival. For temperature seasonality (bio4), the suitability index gradually declines with increasing seasonal temperature variation. The index remains relatively high when temperature seasonality is below 600°C, suggesting that *F. altissima* is poorly adapted to regions with extreme temperature fluctuations, such as northern temperate zones.

The response curve for the clay fraction in fine-grained soils shows a unimodal relationship: the suitability index initially increases and then decreases with rising clay content, peaking at 450 g/kg. This highlights the species’ preference for soils with balanced texture properties, where excessive clay or silt/gravel fractions reduce habitat suitability.

### 3.7 Analysis of current suitable habitats for ancient *F. altissima* under climatic conditions

**Figure 15:**
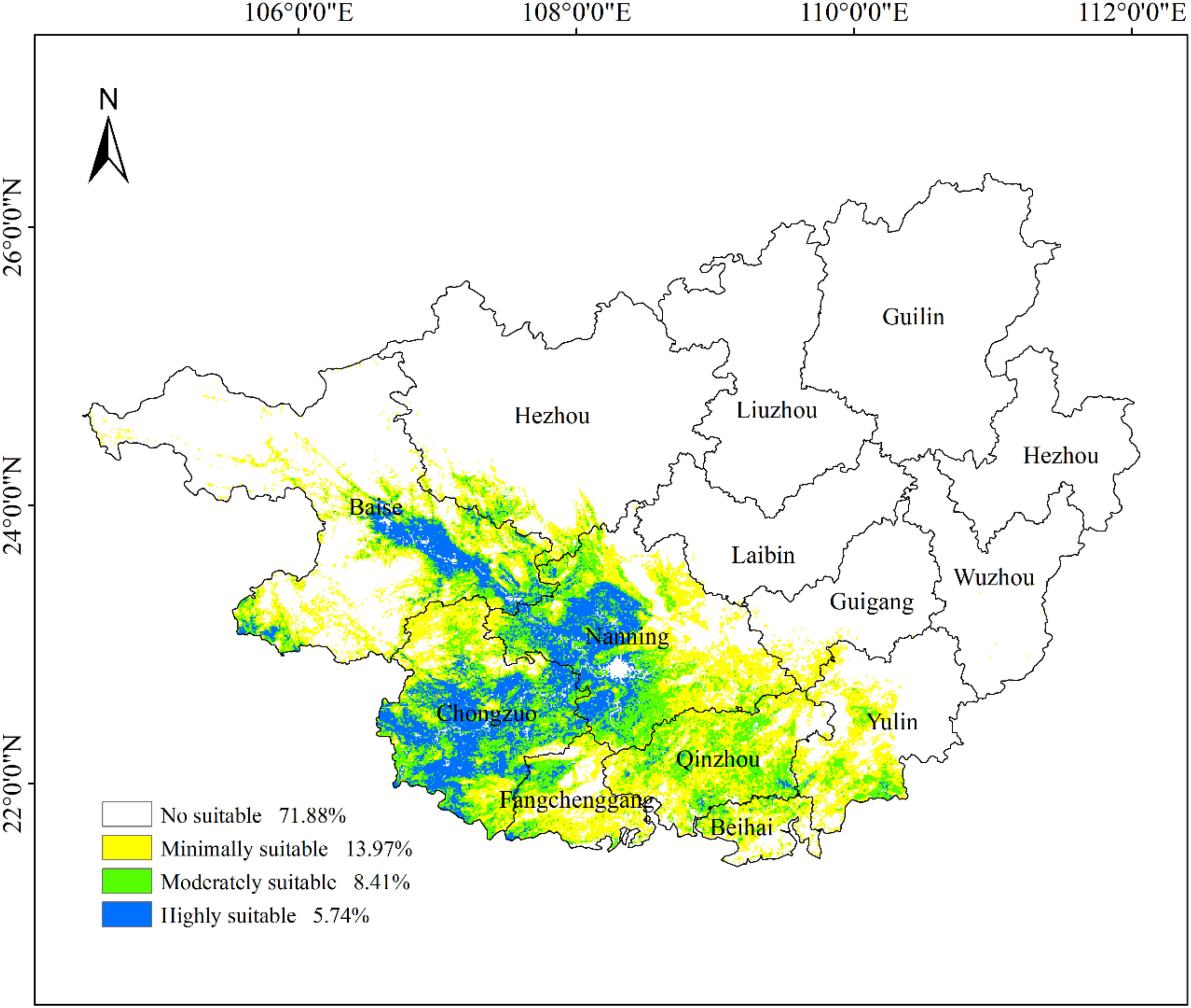
Potential habitat suitability map of *F. altissima* ancient trees.

Using the MaxEnt model and the Jenks natural breaks classification method in ArcGIS 10.8, potential habitat suitability for ancient *F. altissima* in Guangxi Zhuang Autonomous Region was classified into four categories: non-suitable, low-suitability, moderate-suitability, and high-suitability zones (**Figure 15**). The area of each suitability class was quantified. Results indicate that the core high-suitability zones are concentrated in southwestern Guangxi, particularly near the Shiwandashan Mountains and Fangchenggang Nature Reserves, where climatic and edaphic conditions (e.g., warm-humid monsoonal climate, well-drained soils) optimally support the species’ growth. The distribution map further reveals the species’ preferences for specific elevational gradients and topographic features, providing spatially explicit guidance for conservation planning and management interventions.

As illustrated in **Figure 15**, the moderate- and high-suitability zones for ancient *F. altissima* are predominantly concentrated in southern and southwestern Guangxi, accounting for 14.15% of the region’s total area (**Table 2**). The core high-suitability zones are primarily distributed in Baise Municipality, Nanning Municipality, and Chongzuo Municipality. These areas are characterized by warm-humid climatic conditions, with mean annual temperatures ranging from 20 to 25°C and annual precipitation between 1,500 and 2,000 mm, which align closely with the species’ ecological requirements. Edaphically, the dominant soil types in these zones are lateritic red soils and mountainous red soils, which exhibit high fertility and optimal drainage capacity, providing ideal substrate conditions for *F. altissima* growth. Topographically, the species favors low mountainous terrain with moderate slope gradients (5–25°) and northeast/southeast aspects, where sufficient solar irradiance and balanced humidity levels further enhance habitat suitability.

**Table 2:** Area statistics of habitat suitability classes.

Class	Area (km²)	Proportion (%)
Unsuitable	170170.7361	71.88
Low-Suitability Area	33068.21098	13.97
Medium-Suitability Area	19901.18683	8.41
High-Suitability Habitat	14135.37725	5.74

Suitable Habitats (Logical Output Value (LOV) >0.085) are predominantly distributed in western and southern Guangxi Zhuang Autonomous Region, accounting for 28.12% of the study area (**Table 3**). Among these, Low-suitability, Moderate-suitability, and High-suitability zones constitute 13.97%, 8.41%, and 5.74% of the total area, respectively. The High-suitability zones are primarily clustered in Chongzuo Municipality, Nanning Municipality, and the southern part of Baise Municipality.

**Table 3:** Prefecture-level cities with major suitable habitats.

Administrative Region High-Suitability Habitat	Area(km²)	Area Proportion (%)	Districts and Counties
Chongzuo City	6530.636	48.012	Urban Area, Daxin County, Fusui County, Longzhou County, Pingxiang City, Ningming County
Nanning City	4799.134	35.282	Northern Urban Area, Wuming District, Long’an County
Baise City	1810.9889	13.314	Southern Urban Area, Tianyang District, Tiandong County, Pingguo City, Napuo County
Fangchenggang City	183.586	1.350	Shangsi County
Beihai City	142.530	1.048	Western Hepu County
Qinzhou City	89.428	0.657	Southern Urban Area
Hechi City	24.137	0.177	Bama Yao Autonomous County
Yulin City	21.616	0.159	Urban Area, Luchuan County

### 3.8 Suitability analysis of ancient *F. altissima* under future climate scenarios

Under future climate scenarios, the suitability of ancient *F. altissima* in Guangxi was evaluated. *F. altissima* SSP1-2.6 emphasizes sustainable development and strict emission reduction policies, while SSP5-8.5 assumes fossil fuel-dependent economic growth, leading to high greenhouse gas emissions. For future climate scenarios, four time periods (2021–2040, 2041–2060, 2061–2080, and 2081–2100) were analyzed. Model accuracy evaluation results (**Supplementary Figures S6–S13**) showed that all model accuracies were above 0.90.

**Figure 16:**
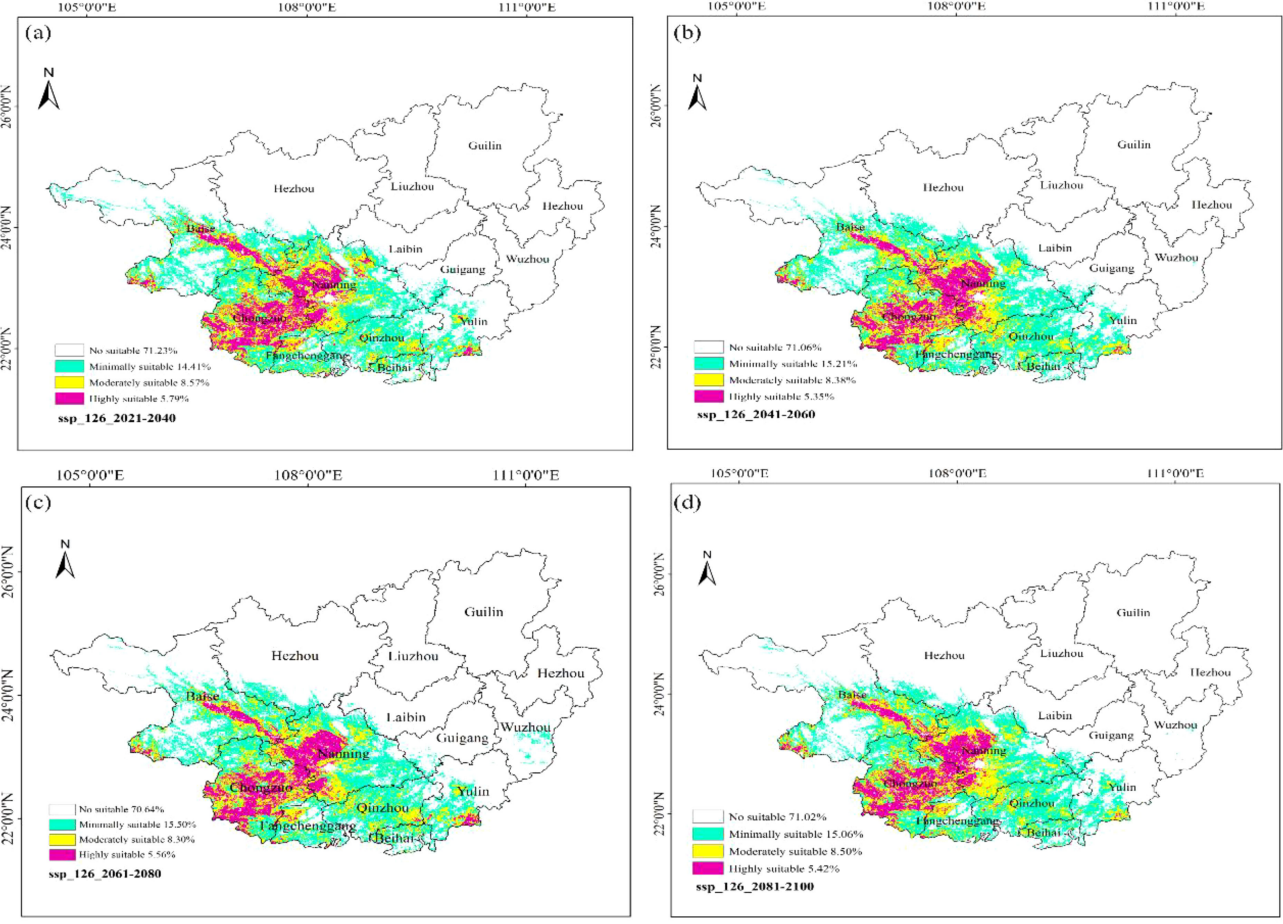
Distribution of suitable habitats for F. altissima in Guangxi Zhuang Autonomous Region under different low-emission scenarios [**(a)** ssp126_2021–2040; **(b)** ssp126_2041–2060; **(c) **ssp126_2061–2080; **(d)** ssp126_2081–2100].

Under the low-emission scenario (SSP1-2.6) (**Figure 16**), the coverage of high-suitability zones accounted for 5.79% of the province during 2021–2040, 5.35% during 2041–2060, 5.56% during 2061–2080, and 5.42% during 2081–2100. The average coverage from 2021 to 2100 was 5.53%. Compared to the current climate data simulation result (5.74%), the distribution range of high-suitability zones showed a slight reduction. The coverage of total suitable habitats (including low, moderate, and high suitability) in the four periods was 28.77%, 28.94%, 29.36%, and 28.98%, respectively, with a multi-year average of 29.01%. Compared to the current coverage (28.12%), the distribution range of suitable habitats for *F. altissima* increased by 0.89%.

**Figure 17:**
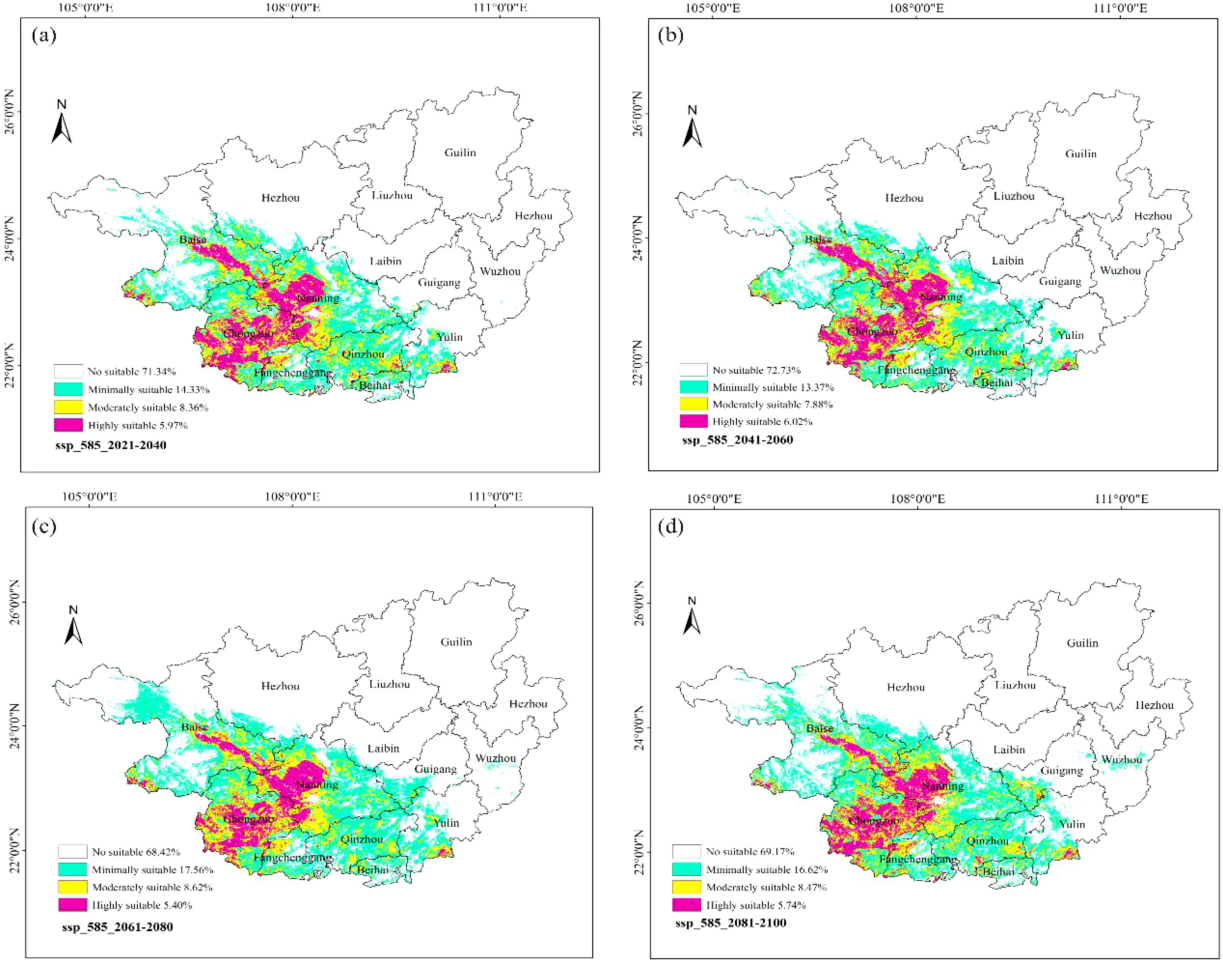
Distribution of suitable habitats for *F. altissima* in Guangxi Zhuang Autonomous Region under high-emission scenarios at different time periods [ **(a)** ssp585_2021–2040; **(b)** ssp585_2041–2060; **(c)** ssp585_2061–2080; **(d)** ssp585_2081–2100].

Under the High-emission Scenario (SSP5-8.5) (**Figure 17**), the high-suitability zones for ancient *F. altissima* accounted for 5.97% of the province during 2021–2040, 6.02% during 2041–2060, 5.40% during 2061–2080, and 5.74% during 2081–2100. The average proportion of high-suitability zones from 2021 to 2100 was 5.78%, reflecting a marginal increase of 0.04% compared to the current baseline (5.74%). However, the total suitable habitat (including low, moderate, and high suitability) across the four periods was 28.66%, 27.27%, 31.58%, and 30.83%, respectively, with a multi-year average of 29.59%. Relative to the current scenario (28.12%), the total suitable habitat increased by approximately 1.47%.

## 4 Discussion and conclusions

### 4.1 Discussion

#### 4.1.1 Distribution pattern of *F. altissima* ancient trees in Guangxi

The tropical and subtropical regions of China constitute the core area of ecological diversity for Ficus species, with their distribution bounded by 28° north latitude (Ding, 2007). Southern provinces such as Fujian, Guangdong, Guangxi, Hainan, and Yunnan serve as the primary habitats for Ficus tree species. Although previous studies have conducted extensive surveys of high-altitude fig trees in the aforementioned regions, most of these studies have been limited to small-scale plant resource surveys and biological statistics (Liu et al., 2013), lacking systematic research on the distribution of ancient high-altitude fig trees throughout Guangxi, particularly in the field of predicting suitable habitats for ancient trees. Based on this, this study innovatively employs the Maximum Entropy (MaxEnt) model and compares it with other mainstream models such as the Bioclim model, MaxNet model, Generalized Linear Model (GLM), Generalized Additive Model (GAM), Natural Splines (NS), and Boosted Regression Trees (BRT). The results indicate that the spatial patterns of suitable habitats predicted by different models are highly consistent. with the suitable habitat of *F. altissima* primarily distributed in the karst terrain regions of central and southern Guangxi. The unique microtopography of these areas may have mitigated some climate change pressures, while the barrier effect of mountain ranges against winter cold fronts and the water-collecting effect of river valley topography provided sustained environmental protection for ancient trees, confirming the important ecological function of karst terrain regions as biological refuge (Yu, 2014; Bátori et al., 2021).

To better protect rare ancient tree resources, this study further explores the evolution patterns of *F. altissima*’s suitable habitats under two climate scenarios. The results indicate that, over time, the total area of suitable habitats under different climate scenarios shows a slight increasing trend. Among these, under the SSP5-8.5 scenario from 2041 to 2060, the high-suitability habitat distribution range of *F. altissima* is the widest, and the area increase is the largest. This finding reveals the positive effect of climate warming on the growth of *F. altissima*, suggesting that moderate warming may promote the expansion of its habitat. Notably, this conclusion contrasts with the current mainstream research perspective: most scholars believe that global climate change will lead to a continuous reduction in species’ suitable habitats, with migration toward higher latitudes or elevations. For example, in 2019, Cao et al. used the MaxEnt model to predict the potential distribution of Qinghai spruce and found that the center of its high-suitability habitat had shifted toward northern high-latitude regions (Cao et al., 2019). In 2022, Wei et al. conducted a study on Perilla frutescens, which indicated that the future suitable habitat area for Perilla frutescens would decrease and shift toward higher latitudes and elevations (Wei et al., 2022). The model prediction results of this study differ from traditional perceptions of climate change, providing new scientific evidence to reveal the specificity of the climate response of ancient trees in the South subtropical region. Model predictions indicate that, in the future, due to warming, the accumulated temperature in the northern hilly areas of Guangxi and the edge of the Yungui Plateau in western Guangxi will increase, which may create new potential suitable habitats for ancient Guangxi Ficus trees. This spatial restructuring process shares similarities with the migration patterns of Ficus species in Yunnan and other regions, reflecting the common response of subtropical tree species to climate change (He, 2015).

#### 4.1.2 Key environmental factors constraining the distribution of ancient *F. altissima* in Guangxi

Empirical research findings demonstrate that the growth performance of ancient trees typically stems from the interplay of various factors (**Table 4**). As shown in **Table 4**, the MaxEnt model is predominantly employed in existing studies to investigate ancient trees. Relatively few studies, however, have demonstrated that individual climatic drivers significantly influence the habitat suitability for ancient tree growth. The MaxEnt model predictions highlight the critical role of specific climatic factors in shaping the potential geographic distribution of ancient *F. altissima* in Guangxi. In this study, temperature variables (bio8, bio4) and the soil factor (clay) emerged as key determinants. Bio8 accounted for 59.1% of the significant contribution, establishing the mean temperature of the wettest quarter as the most influential climatic factor governing the species’ distribution. Similar findings have been reported for other plants, such as *Dipentodon sinicus* (Tang et al., 2020) and *Pterocarpus santalinus* (Zhang et al., 2024b), where the mean temperature of the wettest quarter was also identified as a primary determinant. The highest contribution rate from this temperature variable underscores the species’ heightened demand for solar radiation during the rainy season.

**Table 4:** Key environmental factors simulated by species distribution models for ancient and notable trees.

Old-valuable tree species	Key environmental factors	Model	Study area
*Rucervus eldii eldii* (Anand et al., 2021)	bio3, bio16	MaxEnt	Keibul Lamjao National Park
*Pu’er Tea Trees* (S. Zhang et al., 2022)	bio6, bio12	MaxEnt	Yunnan Province, China
Yulania (Zhang et al., 2024a)	bio2, bio15, DEM	MaxEnt	China
Ormosia microphylla (Wei et al., 2024)	bio2, bio14	MaxEnt	China
Pterocarpus santalinus (Zhang et al., 2024b)	bio4, bio8, bio1, bio12	MaxEnt	China
Ginkgo biloba (Xie et al., 2024a)	bio12	BIOCLIM	China
Keteleeria davidiana (Zhang et al., 2023)	bio4, bio11, bio15	MaxEnt	China
Dipentodon sinicus (Tang et al., 2020)	bio9, bio18, bio8, bio19	MaxEnt	East Asia
Zelkova schneideriana (He et al., 2024)	bio14	MaxEnt	Hunan Province, China
Firmiana kwangsiensis (Gao et al., 2022)	bio18, bio17, Population density, bio12, bio4	MaxEnt	China
All species (Xie et al., 2022)	bio3, bio7	BIOCLIM	Sichuan Province, China
All species (Xie et al., 2020)	bio1, bio5, bio11, bio12, bio14	BIOCLIM	Anhui Province, China

As a critical ecological barrier in southwestern China, Guangxi straddles the transition between southern and central subtropical zones. Monsoonal influences create a distinct environmental regime featuring synchronized hydrothermal patterns and pronounced wet-dry seasonality. Meteorological records indicate mean annual temperatures of 17–23°C, though thermal distributions exhibit three-dimensional heterogeneity: characterized by east-high-west-low gradients and valley-slope differentials (“warm valleys, cool slopes”) resulting from karst topography and maritime moderation. This thermal heterogeneity critically governs vegetation distribution, particularly survival dynamics of relict tree communities.

MaxEnt analysis identified three key drivers of *Ficus altissima* habitat suitability: mean temperature of the wettest quarter (bio8, 59.1%), temperature seasonality (bio4, 18.5%), and soil clay content (4.7%). Current optimal habitats occur in valleys (<300 m) of the Zuojiang and Youjiang basins, where stable bio8 (25–28°C) maximizes photosynthetic efficiency without thermal damage. Existing research data confirm that intense light exposure leads to increased temperature, accelerated transpiration, plant water deficiency, and reduced leaf water potential, which in turn causes a decrease in stomatal conductance (Turner, 1991). Therefore, we need to focus on climate scenarios where bio8 temperature exceeds 29°C. Core habitats maintain bio4 (450–600°C) supporting winter dormancy. Comparative studies demonstrated that in northern Guangxi, where bio4 frequently exceeds 600°C, extreme temperature variability reduces seedling winter survival rates to below 30%. Notably, under the SSP5-8.5 scenario by 2100, bio8 in current core habitats is projected to rise by 2.3–3.1°C, pushing the 28°C isotherm northward and exposing the Zuojiang Basin to thermal stress. However, emerging opportunity zones may arise along the Beibu Gulf coast: despite rising annual temperatures, oceanic moderation limits the bio4 increase to 5–8%, and combined with local clay content exceeding 40%, a new suitable habitat corridor could form between Fangchenggang and Qinzhou. The ecological role of soil clay content is often underestimated, yet it functions as an “invisible regulator”. Soils with 35–45% clay balance water retention capacity and root aeration. However, in exposed bedrock areas, clay content plummets, accelerating rainwater infiltration rates during the wet season and resulting in insufficient water retention—a key reason for the scarcity of *F. altissima* in rocky desertification zones at equivalent latitudes.

#### 4.1.3 Mechanisms of human activity stress on the ecological niche realization of *F. altissima* ancient trees

The ecological niche theory posits that the actual ecological niche of a species is a “dimension-reduced expression” of the baseline ecological niche under biotic and abiotic stress. From the perspective of ecological niche realization, human activities have impacted *F. altissima* ancient trees beyond mere habitat loss, leading to a restructuring of the ecological niche boundaries of these ancient trees. In the karst terrain of Guangxi, the unique “rock-rich, soil-poor” geological characteristics mean that land use changes during urbanization not only severely compress the habitat of ancient trees but also impose stress through ecological niche compression. Firstly, the expansion of farmland or the planting of economic forests (such as fast-growing eucalyptus) in karst areas may alter regional hydrological cycles, leading to decreased groundwater levels or changes in surface runoff, *F. altissima*, as a shallow-rooted species, may have its ability to compete for water resources weakened. Secondly, light pollution and noise interference caused by urbanization alter the activity rhythms of pollinating insects, affecting their symbiotic relationship with *F. altissima* ancient trees; finally, heavy metals (such as Cd and Pb) in household waste accumulate in the root zone (Veeken and Hamelers, 2002), exceeding the tolerance threshold of ancient *F. altissima* trees, leading to physiological metabolic disorders. The synergistic effects of these multiple stresses challenge the “single-factor dominance” assumption in traditional ecological niche theory, providing empirical support for ecological niche reconstruction models under multidimensional stress.

It is worth noting that ancient *F. altissima* trees hold symbolic significance in local culture as “feng shui trees” and “sacred trees,” conferring economic value upon them in tourism development. However, this has also given rise to new threats. In scenic areas or tourist zones, while ancient *F. altissima* trees may gain growth advantages due to management measures, tourism pressures (such as visitor trampling and litter disposal) lead to soil compaction around the roots. The construction of hard surfaces further disrupts material exchange between soil and the atmosphere, inhibiting the decomposition activity of mycorrhizal fungi, ultimately resulting in a decline in soil organic matter content. This indirect impact caused by socio-economic activities alters the soil microenvironment, weakening the *F. altissima*’s nutrient absorption efficiency and conflicting with its ecological niche requirement for fertile soil.

Additionally, introduced non-native species (such as ornamental plants) may compete with *F. altissima* for resources, compressing its ecological niche space. Especially in areas with strong human intervention, such as scenic spots, the synergistic effects of species competition and management measures may reshape the ecological adaptation strategies of ancient *F. altissima* trees.

#### 4.1.4 Conservation and management strategies for ancient *F. altissima* trees

The ancient *F. altissima* trees in Guangxi are “living fossils” of the karst ecosystem, but their survival faces severe challenges due to climate change and human activities. Studies indicate that species with rapidly declining populations are more susceptible to extinction crises (Chichorro et al., 2019). Therefore, the conservation of these ancient trees must not only focus on existing populations but also prepare for future ecological changes. Predictions from the Maximum Entropy Model (MaxEnt) suggest that the current most suitable habitats for *F. altissima* are concentrated in the karst mountainous areas of southwestern Guangxi (e.g., Chongzuo and Jingxi). However, over the next 80 years, rising temperatures and altered precipitation patterns will shift its suitable habitats northward (Chen et al., 2018). In address this trend, it is necessary to implement multidimensional protection measures centered on quantitative indicators and establish a comprehensive framework of “prediction-protection-restoration”.

First, priority should be given to protecting habitats through the implementation of a “thermostable habitat” priority protection strategy. As is well known, biological refuges are areas where species retreat to relatively suitable climatic conditions after their distribution ranges have significantly contracted due to adverse climatic conditions such as cooling and aridity during glacial periods (Zhang et al., 2018). In existing research, commonly used molecular genetic marker methods are limited by sampling areas and typically only allow for qualitative descriptions of species refuges, making precise localization challenging (Waltari et al., 2007). However, when combining species distribution models with ArcGIS software, by calculating the overlapping areas of suitable distributions across multiple climate periods, we can more accurately predict biological refuges.

For changes in suitable areas, high-suitability zones (LOV > 0.48) predicted using the MaxEnt model, combined with climate data from the CMIP6 model under the SSP58.5 scenario, and using ArcGIS to screen for regions where the average annual temperature (bio8) fluctuation is ≤2°C and the seasonal temperature variation (bio4) fluctuation is ≤15% over the next 80 years, these areas are designated as “thermostable core zones” and protected units are delineated at a 1 km × 1 km grid resolution. Additionally, referencing the practices of the Chongzuo Nonggang National Nature Reserve, it is recommended to implement a “one-tree-one-plan” protection scheme for ancient trees with a trunk diameter greater than 1.5 meters at the center of each unit, and establish an ecological buffer zone of 200 meters around them, prohibiting engineering development. For the potential suitable areas in northern Guangxi predicted by the model (such as Luocheng and Huanjiang), a “gravel layer + humus soil” substrate is used to simulate karst rock fissure microhabitats, with seedlings planted at a density of 0.5 plants per square kilometer. Constructing ecological corridors through GIS spatial analysis: Using thermally stable areas as nodes, plant companion tree species such as Quercus acutissima along mountain ranges to form “stepping stone” corridors with a width of ≥2 km, thereby promoting the dispersal of pollinators such as fig bees and maintaining ecological network connectivity (Anstett et al., 1996).

To address the challenges in propagation, urgent conservation of germplasm resources is required. Given the limited natural dispersal capacity of *F. altissima* seedlings, artificial-assisted transplantation is necessary. Based on existing literature data (Huang et al., 2018) and reasonable assumptions, we used the 514 F*. altissima* ancient trees in this study to calculate that, to ensure a 90% survival rate of ancient trees over the next 80 years, when the effective population size ( $N_{e}$ ) is less than 69 trees, we need to avoid inbreeding depression through gene flow ( $N_{m}$ ≥ 0.35) and test the haplotype frequency of *F. altissima* ancient trees every five years. When genetic diversity H_t_< 0.35, it is recommended to artificially introduce individuals from closely related species in Yunnan.

At the same time, by leveraging community resources, the cultural belief in the “dragon vein tree” has been transformed into modern management practices, encouraging residents to voluntarily engage in weed removal and phenological record-keeping activities. In the Yangshuo scenic area, VR technology is used to demonstrate the ecological functions of *F. altissima* ancien trees roots in slope stabilization, enhancing public awareness. In the future, ancient tree protection will require the establishment of an intelligent monitoring network, utilizing drone remote sensing to monitor changes in canopy color to detect pests and diseases early (Stone and Mohammed, 2017; Zhang et al., 2019).

Conserving ancient *F. altissima* trees is akin to restoring a millennia-old ceramic artifact: it requires both preventing further fragmentation of existing structures and identifying compatible new materials to fill gaps. Moving forward, continuous evaluation of the MaxEnt model’s predictive accuracy is essential, with habitat suitability maps updated quinquennially (every five years). It is particularly important to note that climate change may affect ancient tree ecosystems through multiple pathways. Studies have shown that rising temperatures may disrupt the fig-fig wasp symbiotic system, thereby impacting the reproductive and regenerative capacity of ancient trees (Van Kolfschoten et al., 2022). Therefore, when developing conservation strategies, it is essential to consider these ecological interdependencies comprehensively. Ultimately, only through scientific planning and public engagement can these “green umbrellas of stone mountains” continue to safeguard karst landscapes.

#### 4.1.5 Analysis of research limitations and directions for improvement

Due to historical distribution and conservation policies restricting the location of ancient trees, this study is currently unable to achieve the ideal effect of uniformly covering all environmental gradients in the sampling design. The spatial coverage of ancient banyan trees with different health statuses also requires further optimization. Although we employed the commonly used solution in this type of research (ENMTools spatial deduplication), the model’s predicted high-suitability zones may still be influenced to some extent by sampling density. Additionally, soil data only covers 11 conventional indicators such as pH and organic carbon content, and does not include rhizosphere microbial community data. However, existing research has confirmed that mycorrhizal symbiosis is crucial for the stress resistance of ancient trees (Xu et al., 2025).

Future studies could integrate Bayesian network models to combine biotic and abiotic factors, thereby enhancing the ecological interpretability of the model. Karst topography is widely distributed within Guangxi, and rapid drainage caused by karst fissures may limit soil water-holding capacity. The kilometer-scale model we constructed may underestimate the regulatory role of soil texture and microtopography at the local scale. To improve the accuracy of the research results, future studies should include soil profile investigations at the plot scale. Additionally, the model results reflect the theoretical suitable range for *F. altissima*, but actual habitat shifts may lag behind climate warming due to the long reproductive cycle and limited seed dispersal capacity of *F. altissima*. Further investigations could be conducted using population genetics or dispersal models; finally, this study has not yet quantified the direct relationship between socioeconomic factors and ecological niche parameters. Future research could combine remote sensing ecological indices and long-term monitoring data for coupled analysis to more objectively present the study boundaries and highlight the value of subsequent research.

### 4.2 Conclusions

This study employed the multiple species distribution models to delineate potential suitable habitats for ancient *F. altissima* populations in Guangxi Zhuang Autonomous Region and elucidate their key environmental determinants. The Maxent model demonstrates significantly higher AUC and correlation values compared to alternative modeling approaches including NS, BIOCLIM, GLM, and GAM.

Our findings reveal a distinct concentration of high-suitability areas in southwestern Guangxi, characterized by favorable warm-humid microclimates, fertile soils, and optimal drainage conditions. Approximately 62.45% of ancient *Ficus altissima* individuals were distributed at elevations between 80 and 200 meters. We calculated the aboveground biomass of individual ancient trees, with a mean biomass of approximately 27.362 Mg per tree. The tree age of ancient Ficus altissima exhibited a significant correlation with DBH, with a correlation coefficient of 0.64. Tree height was significantly correlated with mean crown width. LOESS regression analysis revealed a statistically significant correlation between tree age and aboveground biomass.

Multivariate analysis identified thermal regime parameters - particularly the mean temperature of the wettest quarter (bio8) and temperature seasonality (bio4) - as the predominant climatic drivers, underscoring the species’ pronounced thermosensitivity and the need for targeted thermal habitat conservation. Secondary but significant influences included edaphic factors (soil type and organic content) and topographic slope gradients.

Under the SSP1-2.6 and SSP5-8.5 climate scenarios, the highly suitable habitat area of *F. altissima* ancient trees showed marginal reductions of 0.04% and 0.21% under these respective scenarios. While demonstrating robust predictive performance (AUC = 0.92), several methodological considerations merit discussion. First, model accuracy remains contingent upon input data quality and appropriate ecological variable selection, with potential biases arising from uneven sampling distributions. Second, parameter sensitivity analyses revealed substantial prediction variability across different regularization multiplier settings. Most notably, the current framework does not account for anthropogenic pressures - a critical limitation given the increasing human footprint in the study region.

## Data Availability

The original contributions presented in the study are included in the article/**Supplementary Material**. Further inquiries can be directed to the corresponding author.
