# Supplementary material for: Assessment of suitability evaluation for Ficus altissima blume ancient trees in different climatic environments in Guangxi, China
Source: Front Plant Sci. 2025 Jul 7;16:1613723. doi: 10.3389/fpls.2025.1613723 (PMC12278064; doi:10.3389/fpls.2025.1613723)
Supplement: Supplementary file 1 [file SupplementaryFile1.docx]

Table S1 Climate variable factor

| Variable | Description |
| --- | --- |
| bio1 | Annual mean temperature |
| bio2 | Mean diurnal range |
| bio3 | Isothermality |
| bio4 | Temperature seasonality |
| bio5 | Max temperature of warmest month |
| bio6 | Min temperature of coldest month |
| bio7 | Temperature annual range |
| bio8 | Mean temperature of wettest quarter |
| bio9 | Mean temperature of driest quarter |
| bio10 | Mean temperature of warmest quarter |
| bio11 | Mean temperature of coldest quarter |
| bio12 | Annual precipitation |
| bio13 | Precipitation of wettest month |
| bio14 | Precipitation of driest month |
| bio15 | Precipitation seasonality (coefficient of variation) |
| bio16 | Precipitation of wettest quarter |
| bio17 | Precipitation of driest quarter |
| bio18 | Precipitation of warmest quarter |
| bio19 | Precipitation of coldest quarter |

Table S2 Soil data and topographic indicators

| Variable | Description |
| --- | --- |
| bdod | Bulk density of the fine earth fraction |
| cec | Cation Exchange Capacity of the soil |
| cfvo | Volumetric fraction of coarse fragments |
| clay | Proportion of clay particles in the fine earth fraction |
| nitrogen | Total nitrogen (N) |
| Phh2o | Soil pH |
| sand | Proportion of sand particles in the fine earth fraction |
| silt | Proportion of sand particles in the fine earth fraction |
| soc | Soil organic carbon content in the fine earth fraction |
| ocd | Organic carbon density |
| ocs | Organic carbon stocks |
| dem | elevation |
| aspect | aspect |
| slope | slope |

Table S3 Accuracy evaluation of different models

| Model | NP | NA | AUC | cor |
| --- | --- | --- | --- | --- |
| BIOCLIM | 362 | 500 | 0.848773 | 0.467325 |
| BRT | 362 | 500 | 0.905304 | 0.726159 |
| GAM | 362 | 500 | 0.923718 | 0.565106 |
| GLM | 362 | 500 | 0.230796 | -0.42563 |
| MaxEnt | 362 | 500 | 0.915271 | 0.733682 |
| MaxNet | 362 | 500 | 0.914591 | 0.525556 |
| NS | 362 | 500 | 0.230663 | -0.46611 |


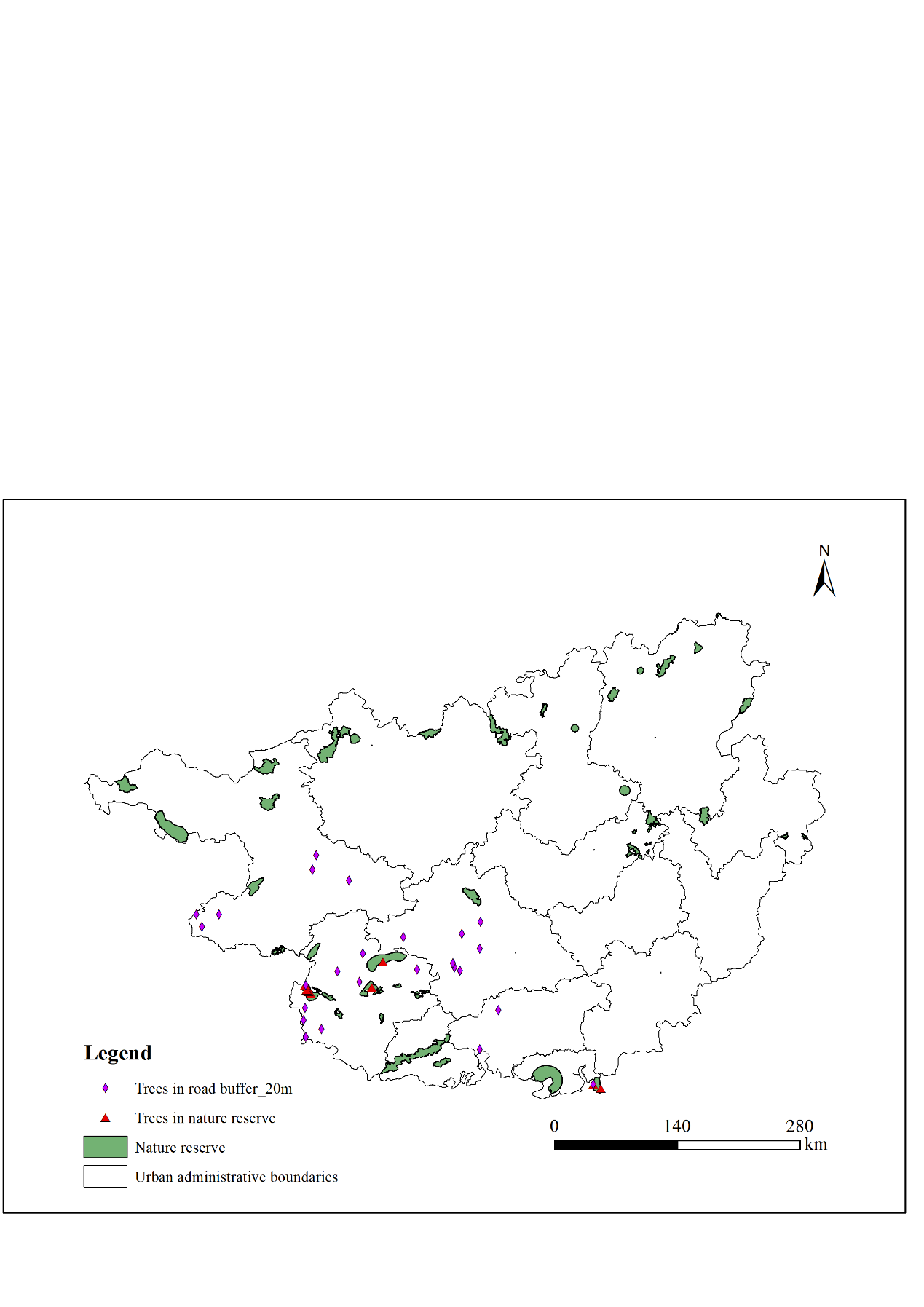


Figure S1. *Ficus altissima* Blume ancient trees in roadside and nature reserve


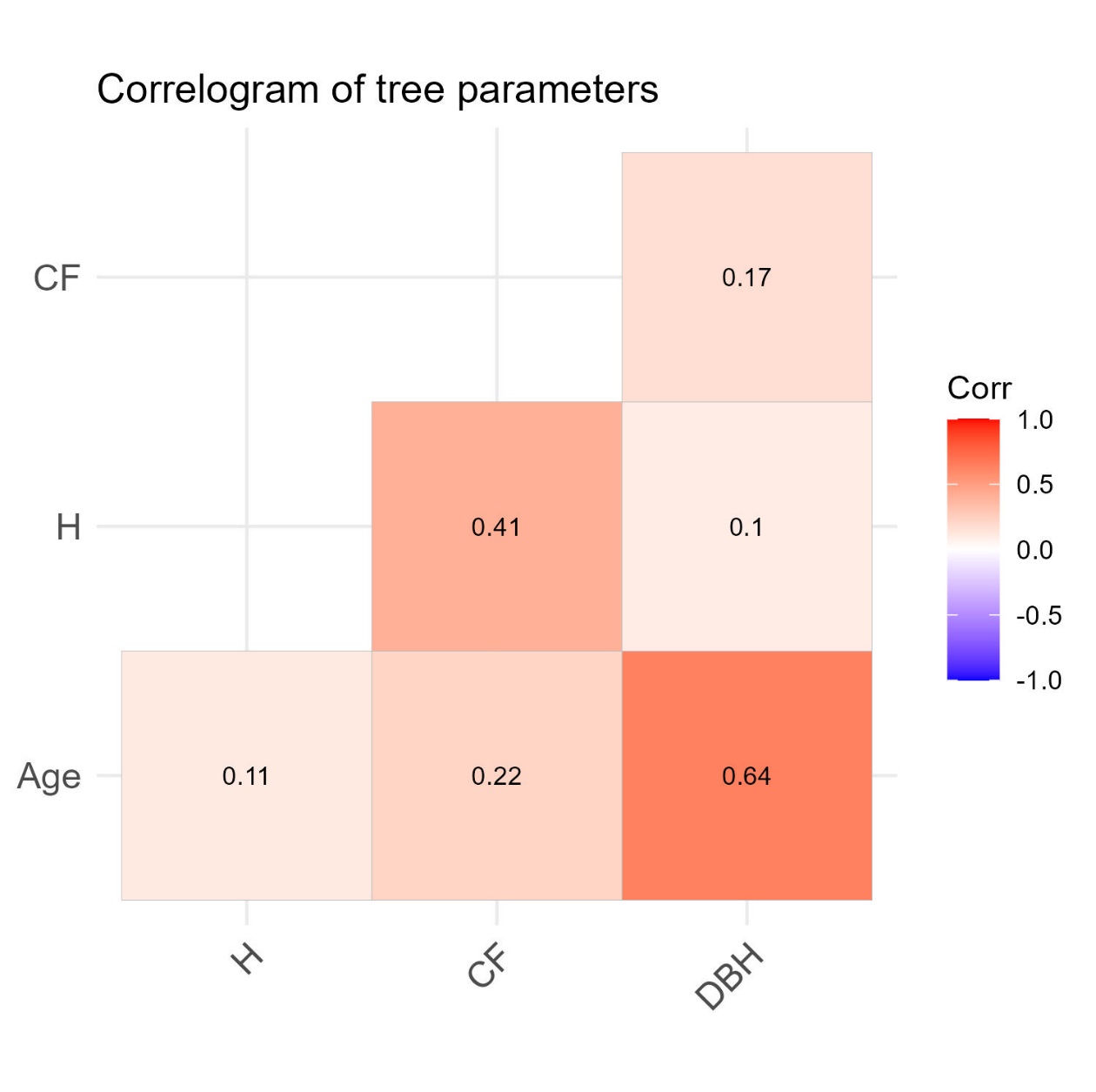


Figure S2. The correlogram of tree parameters (H: tree height, CF: Crown Width, DBH: diameter at breast height)


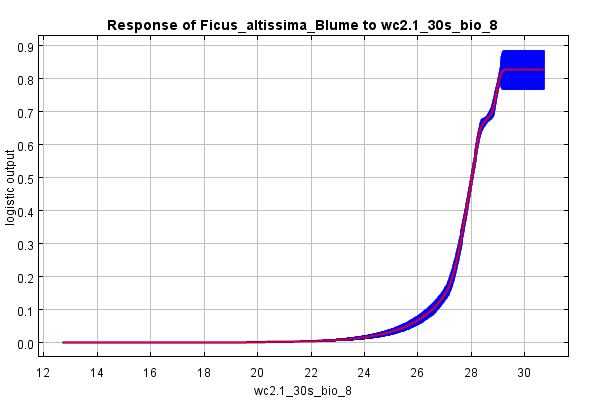
Figure S3. Response curve of modern climate factors (bio8) and changes in suitability index of *Ficus altissima* Blume


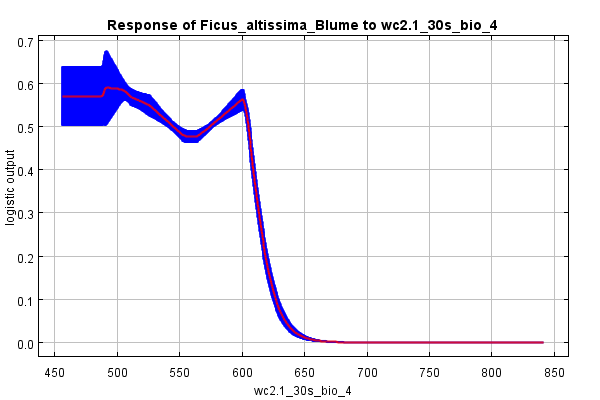


Fgiure S4. Response curve of modern climate factors (bio4) and changes in suitability index of *Ficus altissima* Blume


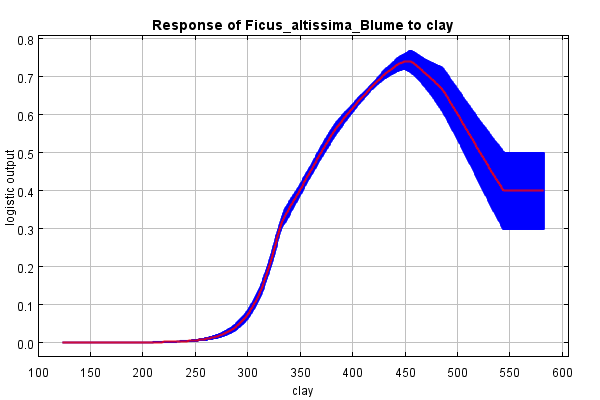


Figure S5. Response curve of modern climate factors (clay) and changes in suitability index of *Ficus altissima* Blume


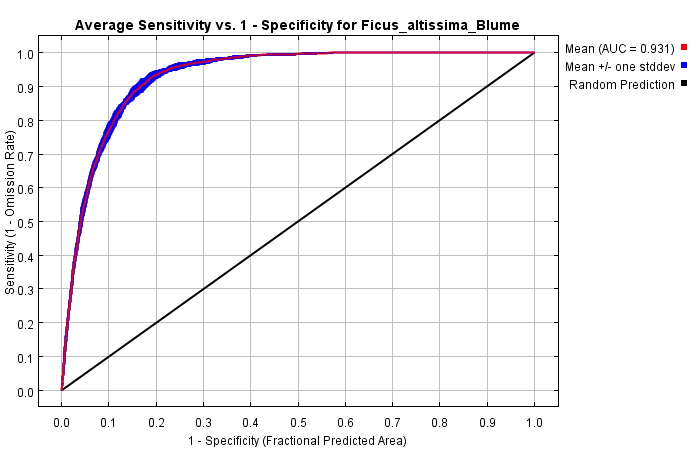


Figure S6. The ROC of the maxent model for ssp126_2021-2040


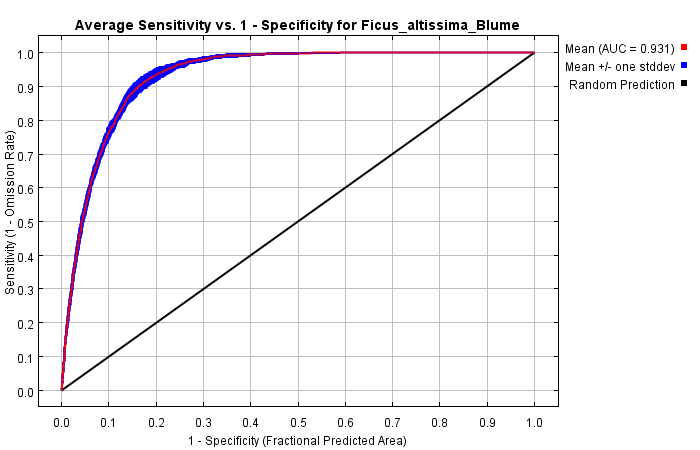


Figure S7. The ROC of the maxent model for ssp126_2041-2060


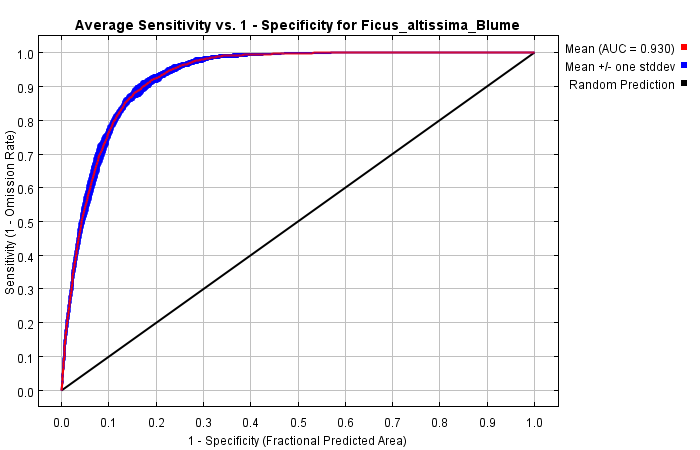


Figure S8. The ROC of the maxent model for ssp126_2061-2080


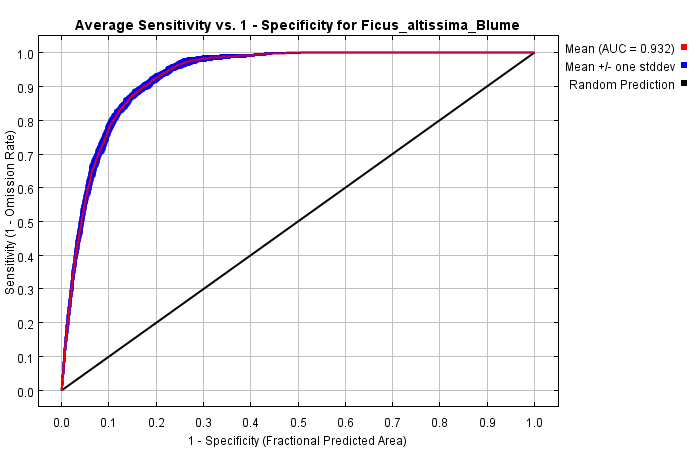


Figure S9. The ROC of the maxent model for ssp126_2081-2100


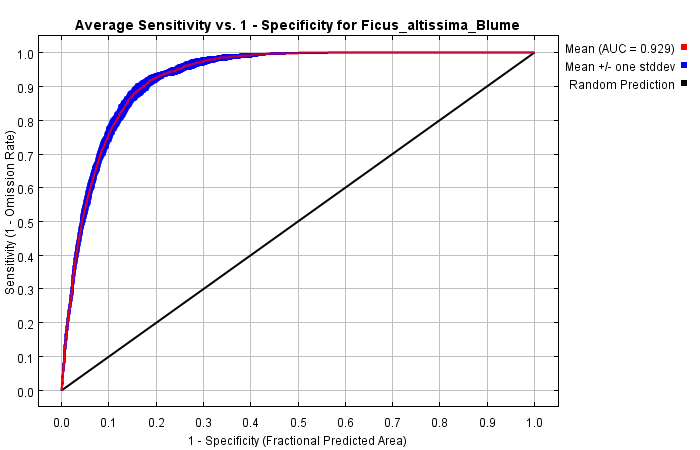


Figure S10. The ROC of the maxent model for ssp585_2021-2040


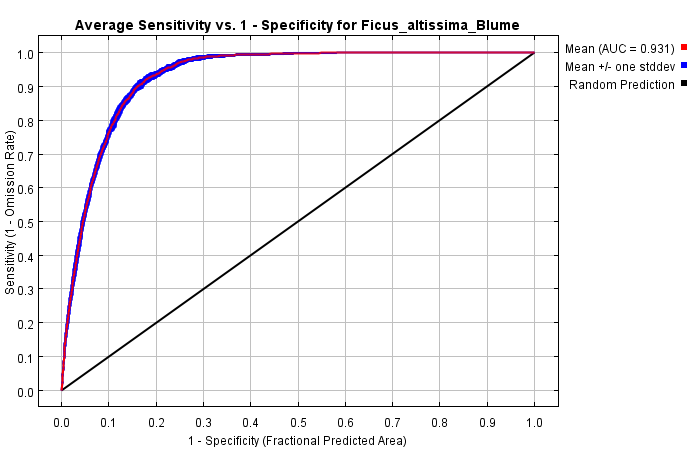


Figure S11. The ROC of the maxent model for ssp585_2041-2060


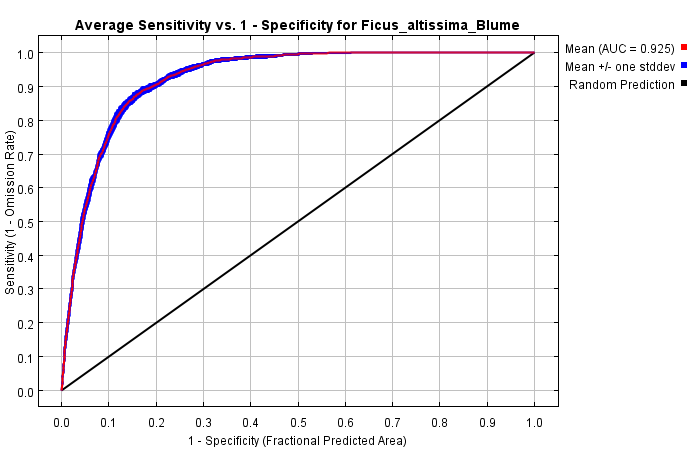


Figure S12. The ROC of the maxent model for ssp585_2061-2080


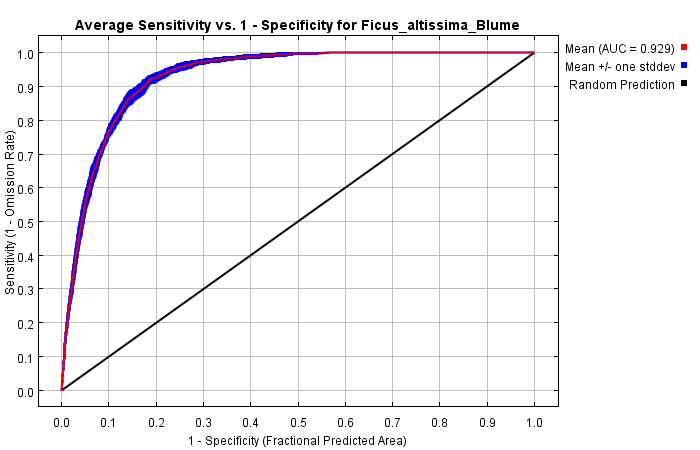


Figure S13. The ROC of the maxent model for ssp585_2081-2100
